# Genome expansion by allopolyploidization in the fungal strain *Coniochaeta* 2T2.1 and its exceptional lignocellulolytic machinery

**DOI:** 10.1186/s13068-019-1569-6

**Published:** 2019-09-23

**Authors:** Stephen J. Mondo, Diego Javier Jiménez, Ronald E. Hector, Anna Lipzen, Mi Yan, Kurt LaButti, Kerrie Barry, Jan Dirk van Elsas, Igor V. Grigoriev, Nancy N. Nichols

**Affiliations:** 1 U.S. Department of Energy Joint Genome Institute, Walnut Creek, CA 94598 USA; 2 Bioagricultural Science and Pest Management Department, Colorado State University, Fort Collins, CO 80521 USA; 3 Microbiomes and Bioenergy Research Group, Department of Biological Sciences, Universidad de los Andes, Carrera 1 No 18A-12, Bogotá, Colombia; 4 Bioenergy Research Unit, National Center for Agricultural Utilization Research, USDA-ARS, Peoria, IL 61604 USA; 5 Cluster of Microbial Ecology, Groningen Institute for Evolutionary Life Sciences, University of Groningen, Nijenborgh 7, 9747 AG Groningen, The Netherlands; 6 Department of Plant and Microbial Biology, University of California Berkeley, Berkeley, CA 94720-3102 USA

**Keywords:** *Coniochaeta*, Fungal genomics, Allopolyploidization, Lignocellulolytic enzymes, Lytic polysaccharide monoxygenases, Wheat straw

## Abstract

**Background:**

Particular species of the genus *Coniochaeta* (Sordariomycetes) exhibit great potential for bioabatement of furanic compounds and have been identified as an underexplored source of novel lignocellulolytic enzymes, especially *Coniochaeta ligniaria*. However, there is a lack of information about their genomic features and metabolic capabilities. Here, we report the first in-depth genome/transcriptome survey of a *Coniochaeta* species (strain 2T2.1).

**Results:**

The genome of *Coniochaeta* sp. strain 2T2.1 has a size of 74.53 Mbp and contains 24,735 protein-encoding genes. Interestingly, we detected a genome expansion event, resulting ~ 98% of the assembly being duplicated with 91.9% average nucleotide identity between the duplicated regions. The lack of gene loss, as well as the high divergence and strong genome-wide signatures of purifying selection between copies indicates that this is likely a recent duplication, which arose through hybridization between two related *Coniochaeta*-like species (allopolyploidization). Phylogenomic analysis revealed that 2T2.1 is related *Coniochaeta* sp. PMI546 and *Lecythophora* sp. AK0013, which both occur endophytically. Based on carbohydrate-active enzyme (CAZy) annotation, we observed that even after in silico removal of its duplicated content, the 2T2.1 genome contains exceptional lignocellulolytic machinery. Moreover, transcriptomic data reveal the overexpression of proteins affiliated to CAZy families GH11, GH10 (endoxylanases), CE5, CE1 (xylan esterases), GH62, GH51 (α-l-arabinofuranosidases), GH12, GH7 (cellulases), and AA9 (lytic polysaccharide monoxygenases) when the fungus was grown on wheat straw compared with glucose as the sole carbon source.

**Conclusions:**

We provide data that suggest that a recent hybridization between the genomes of related species may have given rise to *Coniochaeta* sp. 2T2.1. Moreover, our results reveal that the degradation of arabinoxylan, xyloglucan and cellulose are key metabolic processes in strain 2T2.1 growing on wheat straw. Different genes for key lignocellulolytic enzymes were identified, which can be starting points for production, characterization and/or supplementation of enzyme cocktails used in saccharification of agricultural residues. Our findings represent first steps that enable a better understanding of the reticulate evolution and “eco-enzymology” of lignocellulolytic *Coniochaeta* species.

## Introduction

Species of the genus *Coniochaeta* (phylum Ascomycota; subphylum Pezizomycotina; class Sordariomycetes) have been isolated mainly from furfural-contaminated soil [1], decomposing wood in a mangrove area [2], decaying Acacia trees [3], *Vitis vinifera* plants [4], and soil-derived consortium cultivated on heat pretreated grass [5]. This fungus can switch between a multicellular hyphal form and unicellular yeast growth, depending on environmental and/or nutritional conditions, similar to other reported dimorphic fungi [6]. The asexual phase (i.e., anamorph) of *Coniochaeta* has been classified as *Lecythophora,* and to date, only three draft genome sequences of *Coniochaeta*/*Lecythophora* species have been reported. These include *Coniochaeta pulveracea* CAB683 (genome size: 30.0 Mb), *Lecythophora hoffmannii* CBS245.38 (30.8 Mb) and *C. ligniaria* NRRL30616 (42.3 Mb) [7–9]. In particular, *C. ligniaria* has been studied in light of its capacity to remove toxic furanic compounds from plant biomass dilute-acid hydrolysates, facilitating subsequent microbial fermentation of sugars [10]. In conjunction with this trait, *C. ligniaria* can produce and secrete lignocellulolytic enzymes when grown on corn stover, spelt xylan, microcrystalline cellulose, and kraft lignin [2, 11].

Plant biomass is a carrier of energy with high relevance both ecologically and for biotechnology. Several studies have attempted production of commodity chemicals from agricultural residues [12, 13]. However, one bottleneck in this process is low saccharification efficiency, due largely to the recalcitrant nature of plant polymers [14]. Recently, mining of fungal genomes, transcriptomes, and proteomes has unveiled new enzymes and/or mechanisms that enhance the saccharification of plant polysaccharides [15, 16]. For example, Hüttner et al. [17] and Qin et al. [18] merged genomics and transcriptomics to elucidate the lignocellulolytic machinery in *Malbranchea cinnamomea* (thermophilic ascomycete) and *Irpex lacteus* (white-rot basidiomycete), respectively. Currently, the saccharification process is carried out using commercial enzyme cocktails obtained from *Trichoderma reesei* strains [19]. It has been reported that the supplementation of exogenous enzymes (or secretomes) to *T. reesei*-derived cocktails can improve the saccharification efficiency [20, 21]. Moreover, Harris et al. [22] showed that co-expression of a lytic polysaccharide monoxygenase (LPMO) in a commercial *T. reesei* strain resulted in enhancing conversion of plant biomass. LPMOs (e.g., CAZy families AA9, AA11, AA13, and AA16) are metalloenzymes that catalyze the oxidative cleavage of (1,4)-linked glycosidic bonds of plant polysaccharide surfaces [23]. These proteins have been identified and characterized in several fungal species (e.g., *Neurospora crassa*, *Podospora anserina*, *Thielavia terrestris,* and *Myceliophthora thermophila*) [24, 25]. However, their presence and function in *Coniochaeta* species have yet to be explored.

In this study, we analyzed the genome and transcriptome of *Coniochaeta* sp. strain 2T2.1 to identify its lignocellulolytic machinery. This fungus was isolated from a heat pretreated wheat straw-degrading microbial consortium, where it plays a key role in the degradation of plant polysaccharides, along with bacteria belonging to the genera *Sphingobacterium* and *Klebsiella* [26, 27]. Through genome sequencing, we discovered that 2T2.1 experienced a massive genome duplication event. Changes in genome size have been observed occasionally across members of the Ascomycota and can be caused by several processes including: transposable element expansion spontaneous changes in ploidy, allopolyploidization and autopolyploidization. These last events can hypothetically result in whole-genome duplication (WGD) [28–31]. WGD has the potential to increase fitness for specific functions through diversification of gene function and evolution by selection. Typically, WGD causes genome instability, leading to massive gene loss, genome rearrangements and sequence divergence [32–34]. Consequently, our study sought to answer three main questions: (i) what is the origin of the genome duplication event in 2T2.1? (ii) What lignocellulolytic machinery is present in its genome and how does it differ from other fungal species? (iii) What type of lignocellulolytic enzymes (especially LPMOs) are significantly upregulated during growth on wheat straw compared with glucose? The results of our study expand our “eco-enzymology” (defined here as the study of enzymes and their role in microbial interactions and the modification of surrounding environments) understanding of this fungus and enable the discovery of novel enzymes useful in saccharification of agricultural residues.

## Results

### Morphological and genomic features of *Coniochaeta* sp. 2T2.1

**Fig. 1:**
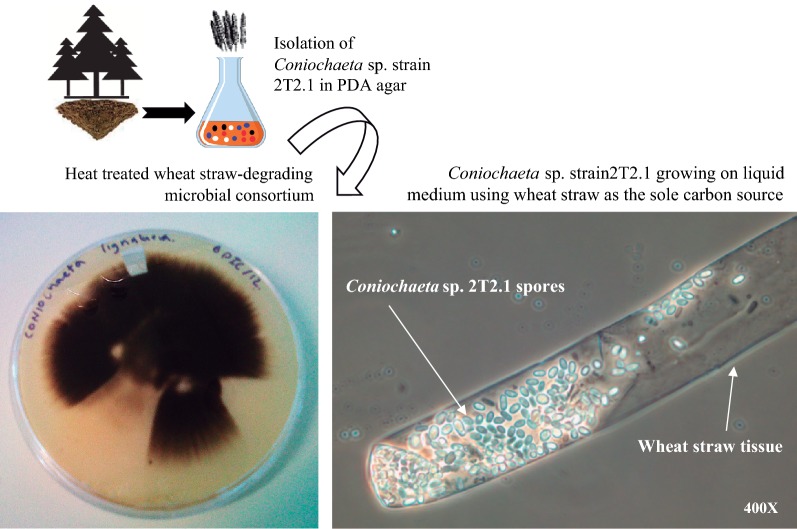
Source of isolation of *Coniochaeta* sp. 2T2.1 [26] and growth on Potato Dextrose Agar (PDA) (left) and in liquid medium using wheat straw as the sole carbon source (micrograph on the right)

On potato dextrose agar (PDA), *Coniochaeta* sp. strain 2T2.1 formed unique black mycelial colonies without evidence of two colony types. In liquid mineral medium supplemented with wheat straw, it grew in a yeast-like form (Fig. 1). The genome of *Coniochaeta* sp. 2T2.1 was sequenced using PacBio technology at the Joint Genome Institute (JGI) and assembled using Falcon, a diploid-aware PacBio assembler [35]. This generated a contiguous, but highly duplicated final assembly with a size of 74.53 Mbp, at read coverage depth of 122.9× with 95 scaffolds larger than 2 Kbp (N50 of 2.67 Mbp and L50 of 11 scaffolds). The three largest scaffolds are all around 4.4 Mb. The proportion of reads with circular intermediates (see methods) that could potentially cause artificial contigs/duplicated content was extremely low (~ 0.3%), indicating that duplicated regions were unlikely to arise due to mis-assembly. Furthermore, junctions between the duplications on the same scaffolds were well supported by PacBio read mapping, indicating a high-quality assembly. The 2T2.1 genome contains 24,735 gene models with an average of 390 amino acids per protein. Around 28% of the total gene models had assigned KEGG functions. From these, some proteins were predicted to be enzymes involved in carbohydrates (1098), amino acids (909), lipids (859), and xenobiotics (806) metabolism. In addition, Pfam domains were located on ~ 67% of genes (16,503 out of 24,735) and ~ 86% (21,299) were supported by transcriptomic data (Additional file 1: Table S1). Other main features of the 2T2.1 genome can be found at JGI-MycoCosm genome portal (https://genome.jgi.doe.gov/Conioc1/).

### Evidence for a genome expansion in *Coniochaeta* sp. 2T2.1

**Fig. 2:**
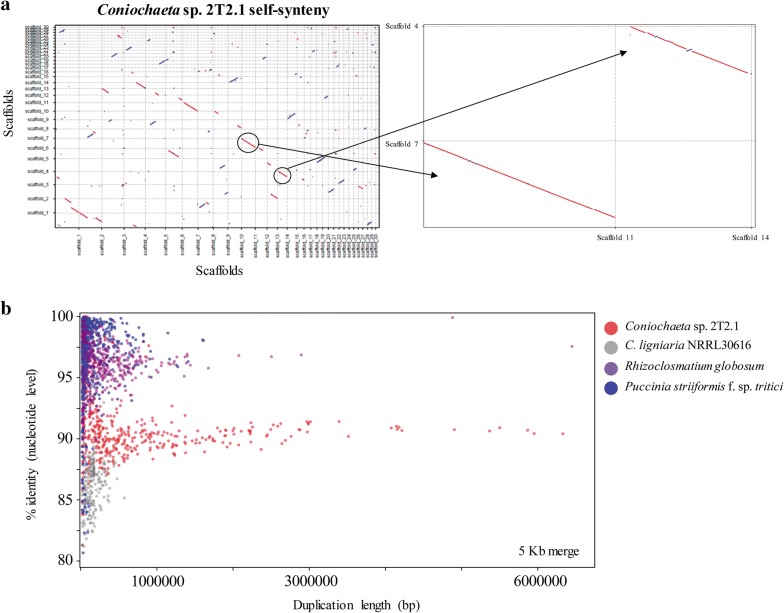
**a** Self-synteny dot plots showing (left) first 30 scaffolds of *Coniochaeta* sp. 2T2.1 and (right) zoom in on two example syntenic regions (scaffold_7:scaffold_11 and scaffold_4:scaffold_14). **b** Length (*x*-axis) and percent identity at the nucleic acid level (*y*-axis) between duplicated regions in *Coniochaeta* sp. 2T2.1 (red) and representative haploid (*C. lignaria*, grey), dikaryotic (*P. striiformis* f. sp. *tritici*, blue) [36] and diploid (*R. globosum*, purple) fungi [37]. Each dot represents a single duplicated region

Unlike other members of the Coniochaetaceae family, strain 2T2.1 displayed a massive genome expansion, resulting in 97.91% of the assembly being duplicated. Duplicated content was identified as regions with at least three genes in each fragment, and at least 50% of genes between fragments were homologous to each other (blastp *e* value ≤ 1e−20 and alignment coverage for both query and target > 80%). This approach revealed that 24,198 (97.83%) of gene models were contained in duplicated regions and 537 genes were found in regions present only once in the assembly. Around 1.55 Mb of the genome is unpaired. For a list of all proteins and their duplication status, see Additional file 2: Table S2. Consistent with genome duplication, much of the assembly is syntenic with other regions in the 2T2.1 genome, although synteny breaks and inversions can be observed (Fig. 2a). To identify the source of this duplication event, we compared genome assembly and gene features to what is typically observed in assemblies of varying ploidy (i.e., haploid, diploid, and dikaryotic lineages). We found that in representative diploid and dikaryotic lineages, over 85% of the total duplicated content was > 95% identical (*Rhizoclosmatium globosum*; diploid: 88.47%, *Puccinia striiformis* f. sp. *tritici*; dikaryon: 88.66%) (Fig. 2b). However, 2T2.1 showed a different pattern from these fungi, as only 2.45% of total duplicated content was > 95% identical. Instead, in 2T2.1, we observed 91.9% nucleotide identity on average (92.33% of duplicated content was between 88.5 and 92.5% identity).

**Fig. 3:**
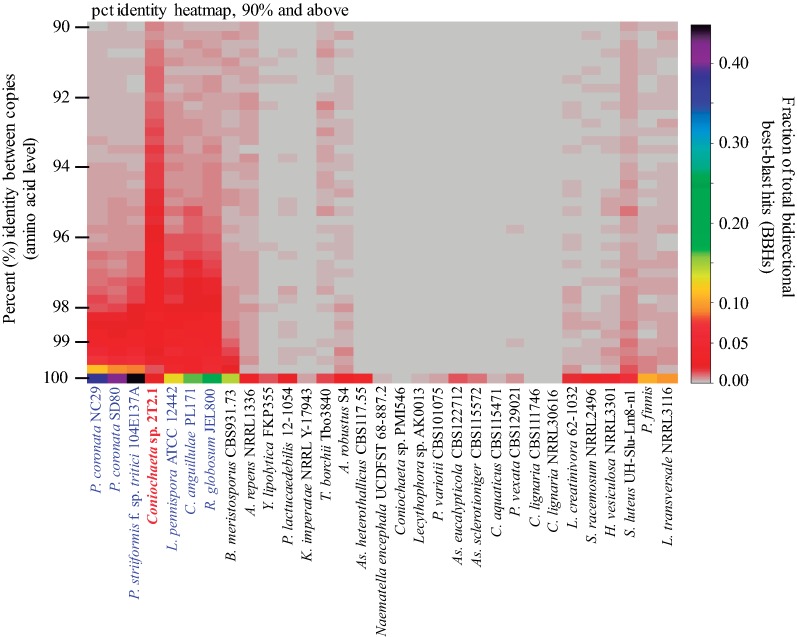
Unique pattern of sequence divergence between duplicates is observed in *Coniochaeta* sp. 2T2.1 (red) compared to haploid (black) and diploid/dikaryotic (blue) fungi. For each genome, a self-BLASTp was conducted to identify duplicates by reciprocal best blast hits (BBHs; min *e* value 1e−5). The fraction of bidirectional best blast hits (BBHs) at varying identity levels (steps = 0.25%) are then plotted (*y*-axis, grey = 0) for each lineage (*x*-axis). Only published PacBio genomes and close relatives of 2T2.1 were included. Despite being dispersed across most of the fungal kingdom, a consistent pattern is observed based on ploidy regardless of phylogenetic neighborhood

Comparing duplicated protein content also shows a dissimilarity of 2T2.1 to patterns observed in other lineages of varying ploidy (Fig. 3; Additional file 3: Fig S1). While allelic proteins from diploid/dikaryotic fungi (labeled in blue in Fig. 3) were frequently > 98% identical to one another, *Coniochaeta* sp. 2T2.1 showed both a higher diversity amongst copies and a depletion of nearly identical copies. For example, in *P. striiformis* (dikaryon), nearly half (44.75%) of all bidirectional best blast hits (BBHs) were 99.75–100% identical in amino acid sequence to each other, while in 2T2.1, this was only 2.46%. Altogether, the features that we observed in 2T2.1 were largely inconsistent with what is typically observed in diploid/dikaryotic assemblies. Since the material for the genome and transcriptome sequencing arose from an isolated colony and only a single mitochondrial sequence was detected, the duplicated content that we observed is unlikely to be due to contamination with a closely related strain.

Therefore, we hypothesized that a whole-genome duplication (WGD) event may have occurred either through (i) a within-species WGD (autopolyploidization) or (ii) recent hybridization of two closely related species (allopolyploidization). However, nucleotide conservation (calculated using nucmer [38]) between 2T2.1 and its closest relatives, genome-sequenced, was substantially lower (*Coniochaeta* sp. PMI546: 85.97% and *Lecythophora* sp. AK0013: 86.73%). Due to the absence of available genomes closely related to 2T2.1, methods such as phylogeny reconstruction [33] are currently unable to resolve whether this duplication occurred through autopolyploidization or allopolyploidization. Furthermore, duplicated genes appear similarly diverged from close relatives, as calculation of synonymous divergence [29, 39] between 2T2.1 duplicates and their orthologues in *Lecythophora* sp. AK0013 did not yield any separation of potential parents (Additional file 3: Fig S2).

**Fig. 4:**
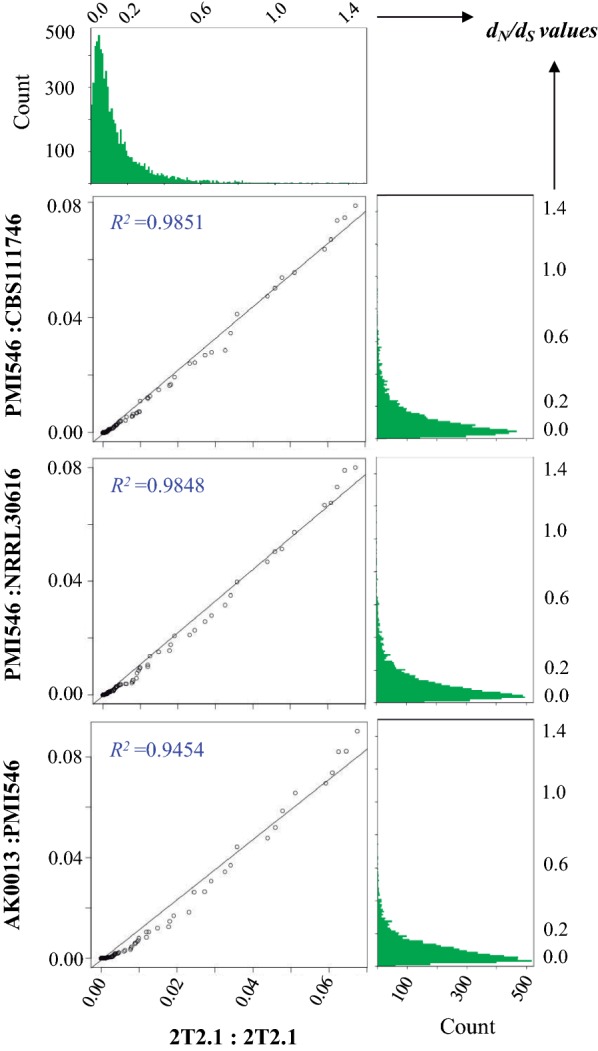
Genome-wide *d*_*N*_/*d*_*S*_ distribution across homeologs in 2T2.1 shows the same distribution as orthologues across species, indicating that the source of this duplication was likely a hybridization event (allopolyploidization). Histograms (green) show *d*_*N*_/*d*_*S*_ distribution across duplicated single-copy genes from *Coniochaeta* sp. 2T2.1 (top left) and single-copy orthologues across: *Coniochaeta* sp. PMI 546 and *C. lignaria* CBS111746 (top right), *Coniochaeta* sp. PMI546 and *C. lignaria* NRRL30616 (middle right), and *Lecythophora* sp. AK0013 and *Coniochaeta* sp. PMI546 (bottom right). Quantile–Quantile plots were then generated to compare *d*_*N*_/*d*_*S*_ distribution in 2T2.1 homeologs with orthologues between species, revealing that distributions are highly correlated (*R*^2^ ≥ 0.945)

Consequently, we developed a different method for separating recent allopolyploidization events from autopolyploidization in 2T2.1. In cases of autopolyploidization, since duplicates are originally at (or near) 100% identity to each other, we expect little or no fitness cost of losing duplicated content (or perhaps even a fitness gain) across most genes in the genome. Therefore, one should observe a rapid accumulation of deleterious mutations and pseudogenization following autopolyploidization, a signature that can be captured by exploring the patterns of nonsynonymous (*d*_*N*_) and synonymous (*d*_*S*_) substitutions across duplicated content. For instance, if copies demonstrate high rates of pseudogenization (*d*_*N*_/*d*_*S*_ ~ 1.0) genome wide, this would suggest autopolyploidization. In contrast, if we observe high rates of purifying selection, this would suggest a recent allopolyploidization, as copies have not coexisted for long enough to accumulate deleterious mutations and become pseudogenes. In the case of *Coniochaeta* sp. 2T2.1, in addition to absence of gene loss despite copies having diverged on average by 8.1% (or 91.9% identity), we observed a strong signature of genome-wide purifying selection. This profile was highly correlated with that observed when comparing single-copy orthologues across different *Coniochaeta*/*Lecythophora* species (*R*^2^ ≥ 0.945; Fig. 4). In other words, the *d*_*N*_/*d*_*S*_ distribution across duplicated genes in 2T2.1 looks the same as between orthologues across species, indicating that the source of the duplication was likely a hybridization event (allopolyploidization) instead of autopolyploidization.

### Clusters of orthologous genes and phylogeny reconstruction

**Fig. 5:**
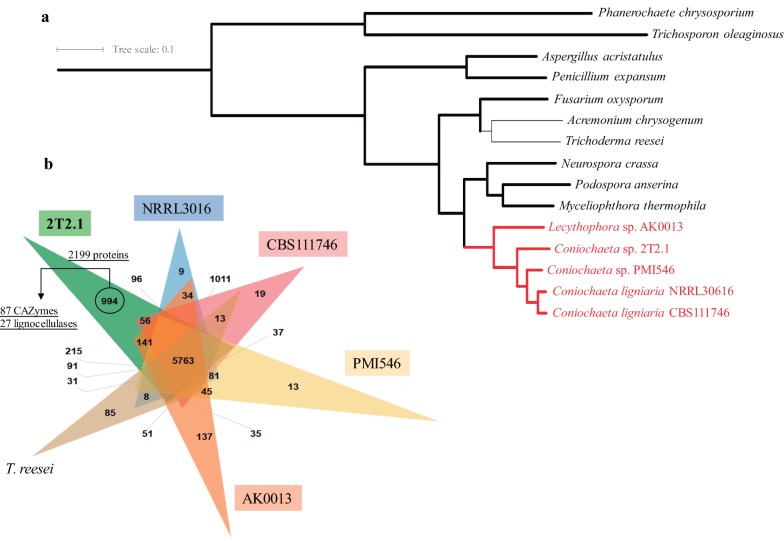
Analysis of orthologous genes. **a** Phylogenetic tree based on 2522 conserved genes (across all genomes) constructed using RAxML. Topology is fully consistent with results from FastTree (Additional file 3: Fig. S3). Fully supported branches are thickened. **b** Number of clusters of orthologous genes within *Lecythophora*/*Coniochaeta* genomes (strains 2T2.1, CBS111746, NRRL30616, PMI546 and AK0013) and *T. reesei* genome. Unique proteins in *Coniochaeta* sp. 2T2.1 (2199) were annotated using the CAZy database

Clusters of orthologous genes were analyzed across the genome of 2T2.1 and those of five other fungi (*C. ligniaria* CBS111746, *C. ligniaria* NRRL30616, *Coniochaeta* sp. PMI546, *Lecythophora* sp. AK0013, and *T. reesei*). A total of 215 and 141 clusters of orthologous genes were shared between 2T2.1 with PMI546 and AK0013, respectively. Moreover, 994 clusters of genes (containing 2199 proteins) were unique in 2T2.1 (Fig. 5b). From these, 87 proteins were affiliated to carbohydrate-active enzymes (CAZymes) and 27 of these were related specifically to lignocellulases (families AA11, AA4, GH43, GH16, GH5, CE1, GH141, GH3, GH31, and CBM16) (Additional file 4: Table S3). For phylogeny reconstruction, we used 2552 single-copy orthologous genes identified using mcl [40] which produced a robust and highly supported tree (RAxML and FastTree) and reveal *Lecythophora* sp. AK0013 as the earliest diverging *Coniochaeta* species that has so far been identified. In addition, *Lecythophora*/*Coniochaeta* species were found to be evolutionarily closer to *N. crassa*, *P. anserina,* and *M. thermophila* than *Fusarium oxysporum*, *T. reesei,* and *Aspergillus chrysogenum* (Fig. 5a; Additional file 3: Fig. S3).

### CAZyme profile of *Coniochaeta* sp. 2T2.1 and comparison with other fungal genomes

From the 24,735 gene models present in 2T2.1, 1376 proteins were predicted to be CAZymes, which we explored deeper to understand the plant biomass-degradation potential of 2T2.1. First, we assessed whether any CAZymes differ significantly (FDR corrected *p* ≤ 0.05; Fisher’s exact test) in abundance in the *Lecythophora*/*Coniochaeta* genomes (2T2.1, CBS111746, NRRL30616, PMI546, and AK0013) compared with the other fungal genomes (Trire2, Neucr_trp3_1, Fusoxy1, Spoth2, Podans1, Aspacri1, Penex1, Acrchr1, Phchr2, and Triol1 (see Additional file 1: Table S1). To make 2T2.1 comparable to other fungi that did not experience a WGD, only one copy was kept for each duplicated gene. Here, we found that the AA8, CBM24, and GH127 families were significantly enriched in the *Lecythophora*/*Coniochaeta* linage.

Next, we determined which gene families from strain 2T2.1 were enriched or depleted (two standard deviations above or below the mean) in abundance in 2T2.1 compared to other fungal genomes. The results showed that genes for lignocellulases from families GH43 (α-arabinosidases/β-xylosidases), GH16 (xyloglucanases/endoglucanases), CE1, CE3 (acetyl xylan esterases), GH11 (endoxylanases), AA4 (vanillyl-alcohol oxidases), and AA1_2 (ferroxidases) were highly abundant in 2T2.1 (more than five genes) compared with the other *Lecythophora*/*Coniochaeta* genomes (Table 1). Moreover, genes for CAZy families CBM24, GH76, CE1, GH47, GH31, GH71, AA8, GH55, AA3, GH11, AA4, AA1_2, AA12, AA3_3, GH13_40, GH45, and GH5_5 were highly abundant in 2T2.1 (more than five genes) compared with the other fungi outside of the Coniochaetaceae. Including all the duplicated content of 2T2.1, the results showed that 122 CAZy families were differentially abundant (two standard deviations above or below) compared to the whole dataset (Coniochaetaceae-derived plus other fungal genomes). Complete counts of all genes belonged to each CAZy family across genomes used in this study (including 2T2.1; Conioc1) is found in Additional file 5: Table S4.

**Table 1:** Comparison of number of CAZymes encoding genes that were differentially abundant (e.g., expansions/contractions) between *Coniochaeta* sp. 2T2.1 (after remove the duplicated content) against other fungal genomes

CAZy family^a^		# of genes in 2T2.1^b^	Mean # of genes in *Lecythophora*/*Coniochaeta* (SD)^c^	Mean # of genes in other fungal genomes (SD)^d^
CBM24		22	X	3.7 (3.4)
GH43	H	22	17.5 (1.5)	X
GH16	H	20	17.0 (0.7)	X
AA3_2		19	10 (0.7)	X
GH76		15	10.25 (0.4)	7.0 (3.7)
CE1	H	13	8.5 (0.8)	4.5 (3.7)
GH47		13	X	7.1 (1.9)
GH31	S	12	X	6 (1.8)
GH71	S	11	X	4.2 (1.5)
AA8		10	X	3.6 (2.2)
GH55		10	X	4.7 (2.6)
AA3		8	4.25 (0.4)	2.1 (2.4)
CE3	H	8	5 (1.2)	X
GH11	H	8	5.75 (0.4)	2.9 (2.3)
AA4	L	7	3.75 (0.4)	1.2 (1.5)
GH32		7	4.5 (0.5)	X
GH78		7	3.75 (0.8)	X
GH7	H	6	8.5 (0.5)	X
AA1_2	L	5	4 (0)	1.5 (1)
AA12		5	X	1.8 (1.3)
AA3_3		5	3 (0.7)	1.6 (0.9)
GH13_40	S	5	X	2.4 (1.2)
GH45	H	5	X	1.2 (0.8)
GH5_5		5	X	2 (1)
CE15	H	4	X	1 (1)
GH128	H	4	5.5 (0.5)	X
GH88		4	1.5 (0.5)	0.8 (1.1)
AA7_dist		3	1 (0)	X
CBM35	H	3	4 (0)	X
CBM52	H	3	X	0.4 (0.6)
GH127	H	3	X	0.4 (0.6)
GH39	H	3	0	0.5 (1.2)
GH5_7		3	4 (0)	X
AA2_dist		2	X	0.4 (0.4)
CE16	H	2	4 (0)	X
GH1	H	2	3 (0)	X
GH114		2	1 (0)	X
GH13	S	2	1 (0)	0.1 (0.3)
GH130		2	0.5 (0.5)	0.1 (0.3)
PL4_1		2	X	0.7 (0.6)

*SD* standard deviation, *X* represented families from 2T2.1 where we not found two standard deviations above or below of the mean counts, *H* predicted (hemi)cellulose-degrading enzymes, *L* predicted lignin-degrading enzymes, *S* predicted starch-degrading enzymes

^a^Only AA, GH, CBM, CE, and PL

^b^Genes with more than two copies after remove duplicated content

^c^Fungal genomes form *C. ligniaria* CBS111746, *C. ligniaria* NRRL30616, *Coniochaeta* sp. PMI546, and *Lecythophora* sp. AK0013

^d^Fungal genomes Trire2, Neucr_trp3_1, Fusoxy1, Spoth2, Podans1, Aspacri1, Penex1, Acrchr1, Phchr2, and Triol1 (see Additional file 1: Table S1)

### Expression of CAZymes by *Coniochaeta* sp. 2T2.1 on wheat straw cultures

We wanted to explore which CAZymes from 2T2.1 may be particularly relevant to lignocellulose degradation through analysis of differential expression (DE) during growth on wheat straw compared with glucose (see methods for details). Therefore, duplicated content was not removed prior to DE analysis. Regarding expression of CAZymes, our result shows that families GH11 (four transcripts), GH10 (three transcripts), CE5, CE1, GH62, GH12, GH51, GH7 (two transcripts from each family), GH93, AA9, CE15, GH127, GH27, GH30, and GH74 (one transcript from each family) were significantly and highly upregulated (padj-value ≤ 0.05, Wald test; and Log2 FC ≥ 10) on raw wheat straw (WS) and dilute-acid-pretreated wheat straw (PTWS) compared with glucose (Glu) cultures. Eight protein-encoding genes from GH11 and seven from GH10 were found in the genome of 2T2.1, indicating that around 50% of these transcripts were overexpressed in WS compared with Glu cultures. In addition, we observed that TPM (transcripts per kilobase million) average values from the most highly upregulated transcripts were even higher in WS compared to PTWS and Glu. Based on the comparison between the FPKM (fragments per kilobase million) values in WS and PTWS vs Glu (FPKM__WS or PTWS_/FPKM__Glu_), we observed that the protein JGI-IDs 1061794 (GH51; α-l-arabinofuranosidase), 961618 (GH62; α-l-arabinofuranosidase), 1273701 (CE5-CBM1; acetyl xylan esterase), 1196733, 1096633 (GH11; endo-β-1,4-xylanase), 1172553 (GH11-CBM1; endo-β-1,4-xylanase), and 1054649 (GH7-CBM1; reducing end-acting cellobiohydrolase) were highly upregulated in both conditions (Table 2).

**Table 2:** Significantly and highly upregulated CAZymes (padj-value ≤ 0.05 and Log2 FC ≥ 10) from *Coniochaeta* sp. 2T2.1 on wheat straw (WS) and pretreated wheat straw (PTWS) compared with glucose (Glu) cultures

JGI-IDs	CAZyme domains	Type of enzyme^b^	FPKM__WS_/FPKM__Glu_	FPKM__PTWS_/FPKM__Glu_	TPM^d^ in WS	TPM^d^ in PTWS	TPM^d^ in Glu
1061794	GH51	α-l-Arabinofuranosidase	71,701	799	1068.13	11.79	0.01
961618	GH62	α-l-Arabinofuranosidase	35,028	262	2435.17	18.27	0.07
1069155	CE1-CBM1	Feruloyl esterase	23,908	1390	1424.70	82.70	0.05
1273638	CE5-CBM1	Acetyl xylan esterase	16,257	1807	1856.79	207.17	0.11
1273701^a^	CE5-CBM1	Acetyl xylan esterase	51,046	10,010	1013.94	199.35	0.02
1196733^a^	GH11	*Endo*-β-1,4-xylanase	32,085	7111	3186.54	705.95	0.10
1172553	GH11-CBM1	*Endo*-β-1,4-xylanase	28,718	2526	1853.9	163.06	0.06
1242067	GH62	α-l-Arabinofuranosidase	638.69^c^	2.32^c^	951.47	3.46	0
955194	GH10-CBM1	*Endo*-β-1,4-xylanase	19,212	1272	572.4	38.06	0.02
1005138	GH10-CBM1	*Endo*-β-1,4-xylanase	14,003	1698	1390.73	168.76	0.09
1231977	GH11-CBM1	*Endo*-β-1,4-xylanase	9612	1190	1241.06	153.69	0.12
344640	GH51	α-l-Arabinofuranosidase	5111	75	812.27	11.94	0.15
1206532	GH62-CBM1	α-l-Arabinofuranosidase	13,418	583	1066.11	46.29	0.07
539071	CE1-CBM1	Feruloyl esterase	2770	65	935.49	22.03	0.33
1096633^a^	GH11	*Endo*-β-1,4-xylanase	10,340	8725	616.15	520.15	0.06
953908	GH12	Endoglucanase	9423	48	327.55	1.69	0.03
970254	GH12	Endoglucanase	3973	135	1282.40	43.43	0.31
382788	GH93	*Exo*-α-l-1,5-arabinanase	2440	19	1175.6	9.48	0.47
980755	AA9	Lytic polysaccharide monoxygenases	102.06^c^	33.52^c^	152.04	50.20	0
969860	CBM1-CE15	4-*O*-Methyl-glucuronoyl methylesterase	3212	519	143.54	23.22	0.04
645961	GH10	*Endo*-β-1,4-xylanase	2013	160	549.92	43.82	0.27
1207935	GH127	β-l-Arabinofuranosidase	2109	62	115.2	3.42	0.05
1265978	GH27-CBM35	α-Galactosidase	32.82^c^	0.85^c^	48.89	1.26	0
1186025	GH30_5	*Endo*-β-1,6-galactanase	60.49^c^	8.71^c^	90.11	12.94	0
1273538	GH74-CBM1	Xylo-endoglucanase	25.48^c^	8.43	37.97	12.60	0
646743	GH7-CBM1	Reducing end-acting cellobiohydrolase	2035	1322	1779.11	1154.39	0.85
1054649^a^	GH7-CBM1	Reducing end-acting cellobiohydrolase	2332	2006	787.48	678.04	0.33

^a^Transcripts that were significantly and highly upregulated in PTWS compared with Glu. Transcripts in this table were significantly and highly upregulated in WS compared with Glu

^b^Putative activity deduced from top-ten hits in BLASTp search against NCBI-nr database

^c^Transcripts that showed values equal to zero in glucose. Here, we showed the FPMK average values in WS or PTWS

^d^Average of transcripts per kilobase million

### Expression of LPMOs by *Coniochaeta* sp. 2T2.1 on wheat straw cultures

**Fig. 6:**
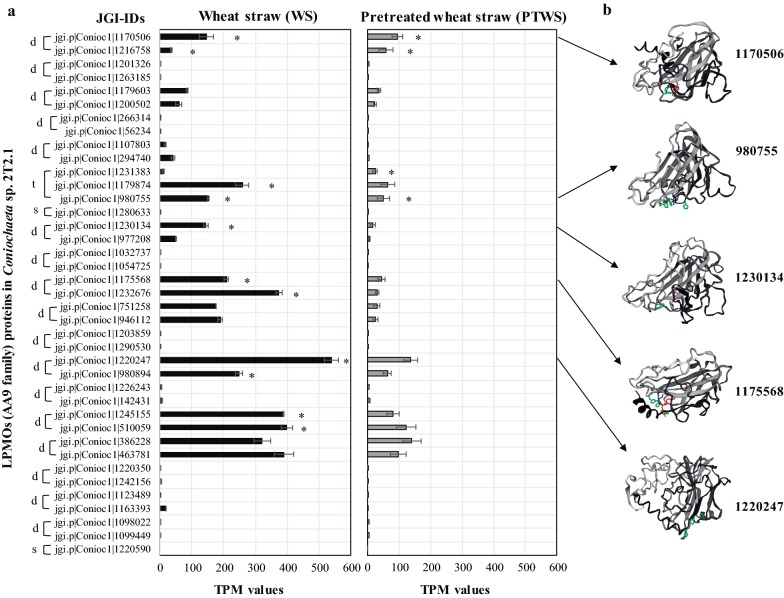
**a** Expression profile (normalized TPM values) of AA9 genes from *Coniochaeta* sp. 2T2.1 after growth (0.1 OD, 600 nm) on wheat straw (WS), and dilute-acid-pretreated wheat straw solids (PTWS). Asterisks represent putative secreted enzymes that were significantly upregulated (padj-value ≤ 0.05 and Log2 FC ≥ 8) in WS and PTWS compared with glucose (Glu) cultures; s, d and t letters represent single, duplicate and triplicate genes within the 2T2.1 genome. **b** Structural 3D modeling of five selected AA9 proteins that were significantly and highly upregulated (padj-value ≤ 0.05 and Log2 FC ≥ 8) on wheat straw (WS) compared with glucose (Glu) cultures. Phyre2 [41] and EZmol [42] web portals were used to predict the putative 3D structural conformation. The molecular size of these proteins (JGI-IDs 1170506, 980755, 1220247, 1175568, and 1230134) ranged between 22 and 29 kDa with different isoelectric points (from 4.56 to 7.51). We identified predicted metal-binding and histidine brace sites based on the structural position and comparison with the best protein for modeling (Additional file 7: Table S6). In the five AA9 proteins, these sites were identified and contain generally two to three histidines (green), one to two tyrosines (red) and one residue of glutamine (blue)

In the 2T2.1 genome, we identified an abundance of genes encoding LPMOs, including 39 from the AA9 family, 8 from AA11, and 2 from AA13. To better understand relationships amongst AA9 proteins, we performed a phylogenetic reconstruction using all 39 proteins from 2T2.1 and AA9 proteins from *C. ligniaria* NRRL30616 (Conlig1), *T. reesei* (Trire2), *P. anserina* (Podans1), and *Phanerochaete chrysosporium* (Phchr2) genomes. Our results revealed that 2T2.1 contains 20 genes encoding family AA9 enzymes, from which, 17 were duplicated, two are unique, and one gene was triplicated or quadruplicated followed by a single gene loss (Additional file 3: Fig. S4). Of these 39 AA9-encoding genes, 11 were significantly upregulated (padj-value ≤ 0.05 and Log2 FC ≥ 8) in WS and 4 were significantly upregulated in PTWS compared with Glu. Those upregulated in WS include four duplicated genes (JGI-IDs 1170506 and 1216758; 1175568 and 1232676; 1220247 and 980894; 1245155 and 510059), two transcripts from a triplicated gene (JGI-IDs 1179874 and 980755) and one transcript from a duplicated gene (JGI-ID 1230134) (Fig. 6; Additional file 3: Fig. S4). In addition, we observed that all significantly upregulated transcripts in WS showed higher TPM values compared with PTWS. The FPKM (FPKM__WS or PTWS_/FPKM__Glu_) and Log2 FC values allowed detection of the top-five AA9 transcripts that were highly upregulated in WS and PTWS compared with Glu (Table 3). Regarding AA11 and AA13 genes, we observed that four and two transcripts, respectively, were significantly upregulated (padj-value ≤ 0.05 and Log2 FC ≥ 2) in WS compared with Glu (Additional file 6: Table S5).

**Table 3:** Log2 FC and normalized expression values (FPKM) of the significantly and highly expressed proteins from CAZy family AA9 (LPMOs) in WS and PTWS compared with Glu

JGI-IDs	Log2 FC (WS vs Glu)	Log2 FC (PTWS vs Glu)	FPKM__WS_/FPKM__Glu_	FPKM__PTWS_/FPKM__Glu_
1170506^a^	9.9	9.4	4980	3189
1175568^a^	9.9	7.7	4670	1019
1179874	8.3	6.4	538	135
1216758	8.2	9	23.47^b^	39.48 f
1220247^ac^	9	7.1	786	199
1230134	8.5	5.6	966	116
1231383	7.1	8.2	7.43^b^	16.44 f
1232676^a^	9.1	5.6	1046	87
1245155^c^	8	5.7	360	75
510059^c^	8.5	6.8	535	164
980755^a^	10	8.4	102.06^b^	33.52 f
980894^c^	8.5	6.6	577	148

^a^Are the top-five upregulated proteins in WS and PTWS compared with Glu

^b^Proteins that showed values equal to zero in glucose. Here, we showed the FPMK average values in WS or PTWS

^c^LPMOs with CBM1 domains

## Discussion

Despite their diverse lifestyles, widespread distribution in different environments [1, 43–45], and lignocellulolytic microbial consortia [46, 47], in-depth omics studies within the *Coniochaeta* have yet to be conducted. Here, we reported the first genomic and transcriptomic survey of a strain belonging to this genus. The *Coniochaeta* sp. strain 2T2.1 showed extracellular endoglucanase and xylanase activities [26], similar to what has been reported for other *Coniochaeta* species [3]. Phylogenomic comparison revealed that strain 2T2.1 was closely related with *Coniochaeta* sp. PMI546 and *Lecythophora* sp. AK0013. These latter two strains were isolated from inside healthy roots of *Populus deltoides* (eastern cottonwood) (https://genome.jgi.doe.gov/ConPMI546/) and the interior of the living moss *Pleurozium schreberi* [48]. Strain 2T2.1 displays two main peculiarities: (i) exceptional (diverse and highly abundant) lignocellulolytic machinery and (ii) an unusual genome duplication event. With respect to lignocellulolytic machinery, genes encoding proteins from CAZy families GH43, GH16, CE1, GH11, AA1_2, and AA4 were highly enriched in the genome of 2T2.1 compared with other fungal genomes, even after removing the duplicated gene content. With nearly double the number of genes in 2T2.1 compared to related fungi, the enrichment of CAZymes in 2T2.1 is even more substantial (Additional file 5: Table S4).

Glycosyl hydrolases (GHs) are key in the breakdown of internal and external linkages of arabinoxylan and xyloglucan [49], while AA1_2 and AA4 proteins could be involved in conversion of lignin. Moreover, 2T2.1 contains 13 CE1-encoding genes, whereas in the genome of *M. thermophila,* we found only four of these [50]. Fungal acetyl xylan esterases (EC 3.1.1.72) from CAZy family CE1 hydrolyze ester bonds to liberate acetic acid from acetylated arabinoxylan and xylooligosaccharides. It has been reported that these enzymes enhance the hydrolysis of pretreated wheat straw and giant reed (*Arundo donax*) [51]. Moreover, using Fisher’s exact test, we found that genes encoding CAZy family GH127 enzymes were significantly enriched in *Lecythophora*/*Coniochaeta* genomes. These types of enzymes are mostly found in bacteria (e.g., *Bifidobacterium longum*), and many have β-l-arabinofuranosidase activity and can act on pectin, arabinoxyloglucan, and glycoproteins that are widely distributed in plant cell walls [52, 53]. Thus, proteins of the GH127 family could play an important role in plant–fungal interactions within *Lecythophora*/*Coniochaeta* species. In addition, we found that one transcript associated with this family was significantly and highly upregulated on wheat straw compared with glucose cultures.

Regarding the genome duplication, we provide arguments, suggesting that 2T2.1 arose due to a hybridization of two related *Coniochaeta*-like species. Considering (i) the substantial diversity between the duplicated regions (91.9% identity on average; Fig. 2b), (ii) the inability of diploid-aware assemblers to phase haplotypes, and (iii) the higher diversity amongst copies and a depletion of nearly identical ones (Fig. 3), it is unlikely that these patterns emerged due to diploidization/dikaryosis. Regarding dikaryosis, this is even less likely as vegetative dikaryons have not been observed in Ascomycota. Alternatively, if the duplication had been caused by autopolyploidization, over the time, it would take the resulting copies to diverge to the extent we observe we would have expected to see the canonical gene loss and genome rearrangement patterns observed in other fungi (e.g., *Rhizopus delamar* 99-880) [30]. Even in the unlikely event that insufficient time has elapsed for rampant gene loss and rearrangements to occur, we should see elevated rates of pseudogenization given the 8% average divergence between copies, which is also not observed. In contrast, gene content was found to be highly conserved in 2T2.1 and a strong genome-wide consensus of purifying selection across copies was detected, similar to what was seen when comparing single-copy orthologues across different species (Fig. 4). As we would not expect nearly all genes in the genome to persist after autopolyploidization and simultaneously be experiencing purifying selection, these features indicate that the most likely source of this duplication event is a hybridization of two different *Coniochaeta* species (allopolyploidization). In addition, this likely occurred in the very recent past, as minimal gene loss has occurred. Previous studies revealed that highly selective environments could force hyphal fusion between unrelated fungi [54, 55]. Since our strain was isolated from the highly selective wheat straw environment, [26, 27], it is possible that to effectively break down plant biomass, two *Coniochaeta*/*Lecythophora* species were forced to fuse together. Alternatively, it is possible that the hybrid can more aggressively break down lignocellulose and is, therefore, more fit in this environment than either parent alone. Moreover, although we have not explicitly explored sexual reproduction here, we have not observed reproductive structures in 2T2.1 and it contains two copies of the same mating type (MAT 1-2-1) (JGI protein IDs 71119 and 1224076). Based on this evidence, we expect that 2T2.1 is heterothallic (i.e., not self-fertile). However, given the limited sampling of this clade, identifying an opposite-mate closely related enough to 2T2.1 to explore fertility of this hybrid is challenging and remains to be addressed.

Through comparing expression profiles of lignocellulolytic enzymes from 2T2.1 grown on wheat straw (raw and/or pretreated) and glucose, we were able to identify several upregulated enzymes which have potential for plant biomass saccharification processes. Remarkably, some of these were associated with endoxylanases (GH10 and GH11), feruloyl (CE1), and acetyl xylan esterases (CE5), which is consistent with what has been reported in *M. cinnamomea* grown on wheat bran and xylan [17]. Feruloyl esterases (EC 3.1.1.73) are responsible for the disruption of the ester bond in the lignin–ferulate–arabinoxylan complex. They act as auxiliary enzymes that assist other enzymes in gaining access to their site of action and, therefore, are likely key to lignocellulolytic activity [56]. Interestingly, α-l-arabinofuranosidases (GH51 and GH62) were also upregulated on 2T2.1 in wheat straw cultures. These enzymes are predicted to cleave the arabinose side chain into arabinoxylan. Qin et al. [18] reported upregulation of family GH61 enzymes in *I. lacteus* during growth on corn stover, whereas de Gouvêa et al. [16] showed that family GH51 enzymes are upregulated in *Aspergillus fumigatus* when the fungus was grown on steam-exploded bagasse compared with fructose. Moreover, Kolbusz et al. [15] studied the CAZy expression profile of *M. thermophila* during cultivation on different types of complex biomass in comparison with glucose. They reported the overexpression of nine enzymes involved in xylan deconstruction (five GH11, one GH62, one CE1, and two CE5) and seven cellulolytic enzymes (three AA9, two GH7, one GH6, and one GH12). In our study, we observed that five significantly and highly upregulated transcripts were associated with endoglucanases (GH12), cellobiohydrolases (GH7), and LPMOs (AA9). These enzymes may comprise the core of the cellulolytic machinery in *Coniochaeta* sp. 2T2.1. Based on this evidence, we suggest that 2T2.1 contains a complete set of enzymes required for exceptionally powerful lignocellulolytic activity. Based on the TPM data, we suggested that the high expression values in raw (WS) over pretreated wheat straw (PTWS) and glucose could be correlated with the highly complex interactions/bonds of the polysaccharides and lignin found in WS. Therefore, the fungal strategy to breakdown this challenging material might be largely based on increased expression and secretion of specific CAZymes.

Fungal LPMOs were first identified in saccharification experiments using pretreated corn stover [22]. Since their discovery, LPMOs have been included in all modern commercial enzyme cocktails (e.g., Cellic CTec3™) [19, 57]. These copper-dependent enzymes boost the activity of classical GHs and cleave glycosidic bonds in cellulose, xylan, xyloglucan, glucomannan, and starch. In our study, after removing duplicate gene content in the 2T2.1 genome, we identified genes for 26 LPMOs (20 AA9-encoding genes). In the genomes of *C. ligniaria* NRRL30616 and *C. pulveracea* CAB683, 23 and 24 LPMOs were identified [7, 9], respectively, whereas in *I. lacteus,* 17 LPMOs were detected that are potentially involved in stimulating (hemi)cellulose degradation [18]. An average plant biomass-degrading fungus has 10 AA9-encoding genes in its genome. Nevertheless, some fungi possess more than 30 different AA9-encoding genes (e.g., *Chaetomium globosum*), indicating a potentially important role of the LPMOs in their lifestyle [58]. For instance, some species of *Coniochaeta* are plant pathogens that could potentially use LPMOs as pathogenicity factors, similar to what was been reported in the maize pathogen *Colletotrichum graminicola* [59]. LPMOs in *Coniochaeta* species could additionally play a role in the decomposition of organic matter in soils. Several factors may be involved in the amplification and diversification of genes encoding LPMOs in 2T2.1. For instance, preference with respect to electron donor, adaptation to minimize undesirable oxidation events and physiochemical preferences [60].

Based on our transcriptomic analysis, we observed that some AA9-encoding genes were highly and significantly upregulated on WS versus Glu. To start characterization of these key LPMOs, we modeled their 3D structure using fungal-derived reported proteins. It is important to mention that LPMOs have low sequence identity, but share the same fold (immunoglobulin-like β-sandwich structure) [24, 60, 61]. To break (1,4)-linked glycosidic bonds of plant polysaccharide surfaces, LPMOs activate oxygen in a reducing agent–dependent manner, at a copper-containing active site known as the “histidine brace”. Unlike GHs, which have substrate-binding grooves or tunnels, LPMOs position their active site at the center of a flat surface. Based on 3D modeling, we identified these sites within five upregulated LPMOs, suggesting a similar structure and/or function with other fungal LPMOs. Notably, protein 1230134 showed a high percentage of identity (80%) with an AA9 family protein from *M. thermophila* [62]. In addition, the 3D model of protein 1175568 was re-constructed based on an AA9 protein from *T. terrestris* (Additional file 7: Table S6). Finally, it is important to note that our research team has recently developed a method for the genetic transformation of strain 2T2.1 using hygromycin as the selectable marker [63]. This method will be very useful for overexpressing lignocellulolytic enzymes that were detected in this study.

## Conclusions

This study reports genomic and transcriptomic features of *Coniochaeta* sp. strain 2T2.1 isolated from a wheat straw-degrading microbial consortium. Interestingly, this fungus experienced an unusual genome duplication resulting from a recent hybridization event between two closely related species. This phenomenon is hypothesized to increase fitness in plant biomass deconstruction. Based on our results, we confirm that strain 2T2.1 has a very complete potential to degrade plant biomass and we highlight the relevance of some CAZy families in these processes (e.g., GH11, GH10, GH62, GH51, AA9, CE1, and CE5). The data presented in this study enable a better understanding of genomic features and metabolic potential of lignocellulolytic *Coniochaeta* species and identify novel proteins useful in saccharification of agricultural residues.

## Materials and methods

### Isolation of *Coniochaeta* sp. 2T2.1 and DNA/RNA extraction

The *Coniochaeta* sp. strain 2T2.1 was originally isolated on PDA from a lignocellulolytic microbial consortium [26, 27]. After 3–4 days of cultivation (30 °C at 250 rpm) in defined mineral medium (MM) [25 mM KH_2_PO_4_, 25 mM Na_2_HPO_4_, 0.1% (NH_4_)_2_SO_4,_ and 0.1% Hutner mineral base] containing 1% (w/w) ground, autoclaved wheat straw (final pH 6.8), the growth of strain 2T2.1 on the substrate was identified using a BX60 microscope (Olympus Life Science, Waltham, MA, USA) with Nomarski interference contrast (Fig. 1). *Coniochaeta*-like fungi form masses of conidia on hyphae, resulting in a yeast-like appearance in liquid culture. The liquid culture was transferred to a yeast extract–peptone–dextrose (YPD) agar and a single colony was isolated and used for reinoculation. To extract fungal genomic DNA, strain 2T2.1 was cultivated at 30 °C under shaking conditions in 50 ml of YPD broth containing 50 μg/ml kanamycin. Total DNA extraction was performed using the OmniPrep kit for fungi (G-Biosciences, St. Louis, MO). Total RNA was then extracted after growth (OD 600 nm of 1.0) on nine different cultures media and conditions: YPD (aerobic and microaerophilic conditions); YPD containing 1.5% (w/v) agar, yeast–peptone (YP); YP plus 1 M NaCl; MM containing 5 mM furfural, 4 mM HMF, and 3 mM benzaldehyde; MM containing glucose and NH_4_ as a nitrogen source; and MM with NO_3_ as nitrogen source and corn stover dilute-acid hydrolysate. Cell pellets were collected by centrifugation. In cases where 2T2.1 was grown on solid medium, cells were scraped off the plate. Subsequently, cells were suspended in 1.0 ml RNALater solution (Qiagen, Venlo, Netherlands) and stored at − 80 °C. Total RNA was isolated using the Qiagen RNAEasy plant mini kit (Qiagen) followed by DNase digestion, and quantified using the Qubit RNA HS assay (ThermoFisher Scientific, Waltham, MA, USA). RNA quality was also assessed visually using RNA bleach gels. The RNA isolated from the above nine cultures was pooled in equal quantities for use in genome annotation.

### Genome and transcriptome sequencing, assembly, and annotation

For genome sequencing, 5 µg of genomic DNA was used to generate unamplified > 10 Kbp libraries. The sheared DNA fragments were then prepared using Pacific Biosciences SMRTbell template preparation kit. Pacific Biosciences hairpin adapters were ligated to the fragments to create the SMRTbell template for sequencing. The SMRTbell templates were then purified using exonuclease treatments and size-selected using AMPure PB beads. PacBio sequencing primer was then annealed to the SMRTbell template library and sequencing polymerase was bound to them using Sequel Binding kit v2.0. The prepared SMRTbell template libraries were then sequenced on a Pacific Biosystem’s Sequel sequencer using v3 sequencing primer, 1 M v2 SMRT cells, and version 2.1 sequencing chemistry with 1 × 360 and 1 × 600 sequencing movie run times. Filtered sub-read data were then assembled together with Falcon version 1.8.8 [35].

Plate-based RNA sample preparation was performed using TruSeq Stranded mRNA HT Sample Prep Kit. Total RNA starting material was 1 µg per sample and 8 cycles of PCR was used for library amplification. The prepared library was then quantified using KAPA Biosystem’s next-generation sequencing library qPCR kit and run on a Roche LightCycler 480 real-time PCR instrument. The quantified library was then multiplexed with other libraries, and the pool of libraries was then prepared for sequencing on the Illumina HiSeq sequencing platform utilizing a TruSeq paired-end cluster kit, v4, and Illumina’s cBot instrument to generate a clustered flow cell for sequencing. Sequencing of the flow cell was performed on the Illumina HiSeq 2500 sequencer using HiSeq TruSeq SBS sequencing kits, v4, following a 2 × 150 indexed run recipe. The raw fastq file reads were filtered and trimmed using the JGI pipeline and assembled into consensus sequences using Trinity version 2.3.2 [64]. Fungal genome annotation was performed using the JGI pipeline and is available via the JGI-MycoCosm genome portal (http://genome.jgi.doe.gov/Conioc1) [65].

### Analysis of *Coniochaeta* sp. 2T2.1 genome with respect to duplication

To explore the duplication event in *Coniochaeta* sp. 2T2.1, we first identified segmentally duplicated regions. These were selected as duplicated genome fragments with a minimum of three genes in each fragment and at least 50% of genes between fragments being homologs to each other (blastp *e* value ≤ 1e−20 and alignment coverage for both query and target > 80%). As we are unable to assign parents to scaffolds due to potential genome rearrangements and similar divergence of duplicates to close relatives (see below), genes in duplicated regions were assigned “copy 1” and “copy 2” designations based on their alphanumeric position in the assembly (Additional file 2: Table S2). The percent assembly in duplication was then calculated as the total sum length of segmentally duplicated regions divided by the total assembly length. To calculate average similarity of 2T2.1 to close phylogenetic relatives (*Lecythophora* sp. AK0013 and *Coniochaeta* sp. PMI546) and representative lineages of varying ploidy, we used nucmer with default parameters from the mummer version 4.4.0 software package [38] and coordinates for all syntenic regions > 2000 bp were extracted using show-coords parameters -l -o -d -c -r -L 2000 -T. For comparison to assemblies of varying ploidy, potentially repetitive sequences (same position mapping to multiple locations) were removed. Since synteny is sometimes interrupted by unique sequence in one of the two copies, neighboring syntenic regions were extended if interrupted by less than 5 kb of non-syntenic sequence. If extended, % identity was averaged across duplicated regions. % of all duplicated content above 95% identity, or between 88.5 and 92.5% was calculated by dividing the sum length of duplicated content in regions at the specified identity levels by the total length of all duplicated content. Whole-genome DNA synteny for the visualization of duplicated content within in 2T2.1 was calculated using VISTA [66] and is available interactively at https://mycocosm.jgi.doe.gov/vista_embed/?viewMode=dotPlot&organism=Conioc1&?&run=47620-mbZaHOBh&xdset=6678&ydset=6730&cutoff=50. As self-alignment will always generate a diagonal line of synteny across the plot, this is uninformative and is automatically removed by VISTA.

To explore patterns of sequence divergence between duplicates in haploid, diploid/dikaryotic and 2T2.1, we included other published fungal genomes deposited on JGI-MycoCosm genome portal that were sequenced using PacBio [36, 37, 67–73], as well as close relatives of 2T2.1. For each genome, a self-BLASTp was conducted using all predicted proteins prior to removal of duplicates to identify orthologues by reciprocal best blast hits (minimum *e* value 1e−5). While the previous publications already identified *P. coronata* f. sp. *avenae* and *P. striiformis* f. sp. *tritici* assemblies to be dikaryotic [36, 69], diploid PacBio assemblies were identified by: (1) analyzing the fraction of associate bases determined by Falcon [35], where any assembly with > 2% associate bases was considered a potential diploid and (2) calculating the fraction of ‘alleles’ present in each genome, where models were determined to be allelic if a secondary models were detected in regions on smaller scaffolds that were > 95% identical at the nucleic acid level and > 50% of the smaller scaffold was covered by these regions. In all cases included here (*Linderina pennispora* ATCC12442, *Catenaria anguillulae* PL171, and *Rhizoclosmatium globosum* JEL800), the percent of associate bases was > 20%, and correspondingly, > 20% of models were determined to be allelic (*L. pennispora*: 24.72%, *R. globosum*: 30.99%, and *C. anguillulae*: 37.09%), indicating that these assemblies are likely diploid. In contrast, in 2T2.1, the percent of associated bases determined by Falcon was 0.53% and only 18 of the 24,735 models (0.073%) fit our criteria to be considered potentially allelic.

Using mcl-identified orthologous gene clusters (see clustering of orthologous genes and phylogenomic comparisons, below), we further conducted an analysis of *d*_*N*_/*d*_*S*_ across duplicated single-copy genes in 2T2.1. Following a similar approach to Mondo et al. [74], we aligned protein sequences using MUSCLE [75], converted to codon alignments using PAL2NAL [76] and then calculated pairwise *d*_*N*_/*d*_*S*_ using the YN00 model [77] implemented in PAML v4.8 [78]. *d*_*N*_/*d*_*S*_ distributions were similarly calculated between single-copy genes in related pairs of species (*Lecythophora* sp. AK0013 and *Coniochaeta* sp. PMI546, *Coniochaeta* sp. PMI546 and *C. lignaria* CBS111746, *Coniochaeta* sp. PMI546 and *C. lignaria* NRRL30616). To quantify similarities between genome-wide *d*_*N*_/*d*_*S*_ distribution patterns in homeologs of 2T2.1 and orthologues across different species, QQ plot analysis was conducted using the EnvStats v2.3.1 package implemented in R version 3.5.1. The same approach was used when attempting to separate parents through comparing *d*_*S*_ [29, 39] between 2T2.1 duplicates and *Lecythophora* sp. AK0031, where any mcl cluster containing a single member from AK0031 and two copies in 2T2.1 were used. AK0031 was chosen for this analysis as it had the highest nucleotide conservation to 2T2.1 based on nucmer results.

### Clustering of orthologous genes and phylogenomic comparisons

To perform phylogenomic comparisons, we selected 14 fungal genomes (including four from the *Lecythophora*/*Coniochaeta* lineage; and eight other Ascomycota, and two Basidiomycota species) that have been deposited on JGI-MycoCosm genome portal (Additional file 1: Table S1). The filtered protein models of each taxon were downloaded, and clusters of orthologous genes among the five *Lecythophora*/*Coniochaeta* genomes were detected using the software OrthoVenn [79]. Unique clusters of proteins found in the genome of *Coniochaeta* sp. 2T2.1 were then annotated using the dbCAN web server [80]. A species tree of *Coniochaeta* was generated using 2522 orthologous genes identified using mcl [40] that were aligned with MAFFT [81]. mcl clusters can be viewed interactively here: https://mycocosm.jgi.doe.gov/clm/run/Conioc1-Study.2509;zFSsaD?organism=Conioc1. Informative sites for phylogenetic purposes were extracted (1,096,767) from the alignment of each orthologous set using GBLOCKs [82], and then, maximum-likelihood phylogeny was re-constructed using both FastTree [83] and RAxML with (100 bootstrap replicates) [84]. Both phylogeny-reconstruction methods used the gamma rate distribution, WAGF substitution model and resulted in nearly fully supported phylogenies that showed the same topology.

### CAZyme genome profile

Annotation of CAZymes in all the genomes evaluated in this study was performed using a combination of BLAST and HMMER searches conducted against the CAZy database [85]. To avoid an overestimation on the number of CAZymes detected in enriched/depleted in the Coniochaetaceae, we removed secondary duplicated gene copies (see methods section: analysis of *Coniochaeta* sp. 2T2.1 genome with respect to duplication) for each CAZy family. For list of secondary duplicates, see Additional file 5: Table S4. Following family assignment, we identified CAZyme families that differed significantly (FDR corrected *p* ≤ 0.05) in abundance in *Lecythophora*/*Coniochaeta* genomes (*Coniochaeta* sp. 2T2.1, *C. ligniaria* CBS111746, *C. ligniaria* NRRL30616, *Coniochaeta* sp. PMI546 and *Lecythophora* sp. AK0013) compared with other fungal genomes using Fisher’s exact test (two-tailed). To explore additional expansions/contractions in 2T2.1, we also determined which CAZy families from 2T2.1 were two standard deviations above or below of the mean counts compared to other *Lecythophora*/*Coniochaeta* genomes (CBS111746, NRRL30616, PMI546, and AK0013) and the other fungal genomes. The same analysis was also conducted including duplicated content (Additional file 5: Table S4). Moreover, LPMOs from family AA9 were extracted from 2T2.1, *C. ligniaria* NRRL30616 (Conlig1), *T. reesei* (Trire2), *P. anserina* (Podans1), and *Phanerochaete chrysosporium* (Phchr2) genomes and used for phylogeny reconstruction using the protocol listed above (see methods section: clustering of orthologous genes and phylogenomic comparisons). In addition, SignalP v.4.1 [86] was used to detect signal peptide cleavage sites in the AA9 proteins.

### Transcriptomic analysis of *Coniochaeta* sp. 2T2.1 growing on different carbon sources

Strain 2T2.1 was cultivated in triplicate in 50 ml of MM containing either: 1% w/v raw wheat straw (autoclaved and cooled before inoculation) (WS), 1% w/v dilute-acid-pretreated wheat straw solids (PTWS), or 1% w/v glucose (Glu). For cultures containing WS or PTWS, flasks were gently shaken and solids were allowed to settle, and then, the liquid fraction was removed by pipetting. The total RNA was extracted as described above when the cultures reached an optical density of 1.0 (OD 600 nm). Stranded RNAseq libraries were created and quantified by qPCR. RNA sequencing was performed using an Illumina HiSeq HiSeq-2500 1TB 1 × 101 instrument. Using BBDuk (https://sourceforge.net/projects/bbmap/), raw reads were evaluated for artifact sequence by kmer matching (kmer = 25), allowing one mismatch and detected artifact were trimmed from the 3′ end of the reads. RNA spike-in reads, PhiX reads, and reads containing any Ns were removed. Quality trimming was performed using the Phred trimming method set at Q6. Finally, reads under the length threshold were removed (minimum length 25 bases or 1/3 of the original read length—whichever is longer). Filtered reads from each library were aligned to the 2T2.1 reference genome (Conioc1) using HISAT2 version 2.1.0 [87]. HISAT2 searches for up to *N* distinct, primary alignments for each read, where *N* equals the integer specified with the − *k* parameter. Primary alignments mean alignments, whose alignment score is equal or higher than any other alignments. It is possible that multiple distinct alignments have the same score. However, for *Coniochaeta* sp. 2T2.1, we set *k* = 1, meaning that only unique primary alignments were included in downstream analysis. Across all libraries, 97.62% to 99.27% of reads mapped uniquely to the 2T2.1 genome, indicating that duplicated regions were sufficiently diverged to allow accurate read mapping. FeatureCounts [88] was then used to generate the raw gene counts file using gff3 gene models. Only primary hits assigned to the reverse strand were included in the gene counts (Additional file 8: Table S7 contains libraries and raw counts). Raw gene counts were used to evaluate the level of similarity between biological replicates using Pearson’s correlation. DESeq 2 (version 1.18.1) [89] was subsequently used to determine which genes were differentially expressed between pairs of conditions. A table with the Log2 FC (fold change), adjusted pval (padj-value) and whether the gene is significantly and differentially expressed (TRUE/FALSE/NA) for each pair of conditions was then generated. In addition, FPKM (fragments per kilobase million) and TPM (transcripts per kilobase million) normalized gene counts were obtained using the RNAseq gene expression analysis pipeline at the JGI.

## Supplementary information

**Additional file 1: Table S1.** Assembly and annotation statistics from fungal genomes used in this study.

**Additional file 2: Table S2.** List of all proteins found in the 2T2.1 genome and their duplication status.

**Additional file 3.** Additional figures (S1 to S3).

**Additional file 4: Table S3.** Unique CAZymes found in *Coniochaeta* sp. 2T2.1 after comparison with other *Lecythophora*/*Coniochaeta* genomes (NRRL3016, CBS111746, PMI546 and AK0013) and *Trichoderma reseei* genome (Trire2).

**Additional file 5: Table S4.** Number of CAZymes across all genomes evaluated in this study.

**Additional file 6: Table S5.** Expression values of AA11 and AA13 genes from *Coniochaeta* sp. 2T2.1 after growth (0.1 OD, 600 nm) on wheat straw (WS) and dilute-acid-pretreated wheat straw solids (PTWS).

**Additional file 7: Table S6.** 3D modeling features of five selected AA9 proteins that were significantly and highly upregulated (padj-value ≤ 0.05 and Log2 FC ≥ 8) on wheat straw (WS) compared with glucose (Glu) cultures.

**Additional file 8: Table S7.** Transcriptomic raw data (libraries names and raw counts in each library).

## Data Availability

The data sets supporting the findings of this study are included as Additional files 1 to 8. This whole-genome shotgun project has been deposited at DDBJ/ENA/GenBank under the accession VSMA00000000 (BioProject PRJNA250595). The version described in this paper is version VSMA01000000. The transcriptome data were deposited under the following SRA accessions numbers (SRP170777, SRP170791, SRP170792, SRP170785, SRP170784, SRP170782, SRP170783, and SRP170789).

## Abbreviations

LPMOs: lytic polysaccharide monoxygenases
WGD: whole-genome duplication
CAZymes: carbohydrate-active enzymes
MM: mineral medium
YPD: yeast extract–peptone–dextrose
WS: raw wheat straw
PTWS: pretreated wheat straw
Glu: glucose
GH: glycosyl hydrolase
FPKM: fragments per kilobase million
TPM: transcripts per kilobase million
PDA: potato dextrose agar
JGI: Joint Genome Institute

## References

[1] López MJ, Nichols NN, Dien BS, Moreno J, Bothast RJ (2004). Isolation of microorganisms for biological detoxification of lignocellulosic hydrolysates. Appl Microbiol Biotechnol.

[2] Ravindran A, Adav SS, Sze SK (2012). Characterization of extracellular lignocellulolytic enzymes of *Coniochaeta* sp. during corn stover bioconversion. Process Biochem.

[3] van Heerden A, van Zyl WH, Cruywagen CW, Mouton M, Botha A (2011). The lignicolous fungus *Coniochaeta pulveracea* and its interactions with syntrophic yeasts from the woody phylloplane. Microb Ecol.

[4] Casieri L, Hofstetter V, Viret O, Gindro K (2009). Fungal communities living in the wood of different cultivars of young *Vitis vinifera* plants. Phytopathol Mediterranea..

[5] Trifonova R, Postma J, Verstappen FW, Bouwmeester HJ, Ketelaars JJ, van Elsas JD (2008). Removal of phytotoxic compounds from torrefied grass fibres by plant-beneficial microorganisms. FEMS Microbiol Ecol.

[6] Boyce KJ, Andrianopoulos A (2015). Fungal dimorphism: the switch from hyphae to yeast is a specialized morphogenetic adaptation allowing colonization of a host. FEMS Microbiol Rev.

[7] Jiménez DJ, Hector RE, Riley R, Lipzen A, Kuo RC, Amirebrahimi M (2017). Draft genome sequence of *Coniochaeta ligniaria* NRRL 30616, a lignocellulolytic fungus for bioabatement of inhibitors in plant biomass hydrolysates. Genome Announc..

[8] Leonhardt S, Büttner E, Gebauer AM, Hofrichter M, Kellner H (2018). Draft Genome Sequence of the Sordariomycete *Lecythophora* (*Coniochaeta*) *hoffmannii* CBS 24538. Genome Announc..

[9] Borstlap CJ, de Witt RN, Botha A, Volschenk H (2019). Draft genome sequence of the lignocellulose-degrading ascomycete *Coniochaeta pulveracea* CAB 683. Microbiol Resour Announc..

[10] Nichols NN, Dien BS, Cotta MA (2010). Fermentation of bioenergy crops into ethanol using biological abatement for removal of inhibitors. Bioresour Technol.

[11] López MJ, Vargas-Garcia MC, Suarez-Estrella F, Nichols NN, Dien BS, Moreno J (2007). Lignocellulose-degrading enzymes produced by the ascomycete *Coniochaeta ligniaria* and related species: application for a lignocellulosic substrate treatment. Enzyme Microb Technol.

[12] Talebnia F, Karakashev D, Angelidaki I (2010). Production of bioethanol from wheat straw: an overview on pretreatment, hydrolysis and fermentation. Bioresour Technol.

[13] Novy V, Longus K, Nidetzky B (2015). From wheat straw to bioethanol: integrative analysis of a separate hydrolysis and co-fermentation process with implemented enzyme production. Biotechnol Biofuels.

[14] Himmel ME, Ding SY, Johnson DK, Adney WS, Nimlos MR, Brady JW (2007). Biomass recalcitrance: engineering plants and enzymes for biofuels production. Science.

[15] Kolbusz MA, Di Falco M, Ishmael N, Marqueteau S, Moisan MC, Baptista CDS (2014). Transcriptome and exoproteome analysis of utilization of plant-derived biomass by *Myceliophthora thermophila*. Fungal Genet Biol.

[16] de Gouvêa PF, Bernardi AV, Gerolamo LE, de Souza Santos E, Riaño-Pachón DM, Uyemura SA (2018). Transcriptome and secretome analysis of *Aspergillus fumigatus* in the presence of sugarcane bagasse. BMC Genomics..

[17] Hüttner S, Nguyen TT, Granchi Z, Chin-A-Woeng T, Ahrén D, Larsbrink J (2017). Combined genome and transcriptome sequencing to investigate the plant cell wall degrading enzyme system in the thermophilic fungus *Malbranchea cinnamomea*. Biotechnol Biofuels.

[18] Qin X, Su X, Luo H, Ma R, Yao B, Ma F (2018). Deciphering lignocellulose deconstruction by the white rot fungus *Irpex lacteus* based on genomic and transcriptomic analyses. Biotechnol Biofuels.

[19] Johansen KS (2016). Discovery and industrial applications of lytic polysaccharide mono-oxygenases. Biochem Soc Trans.

[20] Gao D, Uppugundla N, Chundawat SP, Yu X, Hermanson S, Gowda K (2011). Hemicellulases and auxiliary enzymes for improved conversion of lignocellulosic biomass to monosaccharides. Biotechnol Biofuels.

[21] Poidevin L, Berrin JG, Bennati-Granier C, Levasseur A, Herpoël-Gimbert I, Chevret D (2014). Comparative analyses of *Podospora anserina* secretomes reveal a large array of lignocellulose-active enzymes. Appl Microbiol Biotechnol.

[22] Harris PV, Welner D, McFarland KC, Re E, Navarro Poulsen JC, Brown K (2010). Stimulation of lignocellulosic biomass hydrolysis by proteins of glycoside hydrolase family 61: structure and function of a large, enigmatic family. Biochemistry.

[23] Horn SJ, Vaaje-Kolstad G, Westereng B, Eijsink VG (2012). Novel enzymes for the degradation of cellulose. Biotechnol Biofuels.

[24] Hemsworth GR, Johnston EM, Davies GJ, Walton PH (2015). Lytic polysaccharide monooxygenases in biomass conversion. Trends Biotechnol.

[25] Vaaje-Kolstad G, Forsberg Z, Loose JS, Bissaro B, Eijsink VG (2017). Structural diversity of lytic polysaccharide monooxygenases. Curr Opin Struct Biol.

[26] Jiménez DJ, Korenblum E, van Elsas JD (2014). Novel multispecies microbial consortia involved in lignocellulose and 5-hydroxymethylfurfural bioconversion. Appl Microbiol Biotechnol.

[27] Jiménez DJ, Dini-Andreote F, van Elsas JD (2014). Metataxonomic profiling and prediction of functional behaviour of wheat straw degrading microbial consortia. Biotechnol Biofuels.

[28] Castanera R, López-Varas L, Borgognone A, LaButti K, Lapidus A, Schmutz J (2016). Transposable elements versus the fungal genome: impact on whole-genome architecture and transcriptional profiles. PLoS Genet.

[29] Ortiz-Merino RA, Kuanyshev N, Braun-Galleani S, Byrne KP, Porro D, Branduardi P, Wolfe KH (2017). Evolutionary restoration of fertility in an interspecies hybrid yeast, by whole-genome duplication after a failed mating-type switch. PLoS Biol.

[30] Ma LJ, Ibrahim AS, Skory C, Grabherr MG, Burger G, Butler M (2009). Genomic analysis of the basal lineage fungus *Rhizopus oryzae* reveals a whole-genome duplication. PLoS Genet.

[31] Harari Y, Ram Y, Rappoport N, Hadany L, Kupiec M (2018). Spontaneous changes in ploidy are common in yeast. Curr Biol.

[32] Albertin W, Marullo P (2012). Polyploidy in fungi: evolution after whole-genome duplication. Proc Biol Sci..

[33] Marcet-Houben M, Gabaldón T (2015). Beyond the whole-genome duplication: phylogenetic evidence for an ancient interspecies hybridization in the baker’s yeast lineage. PLoS Biol.

[34] Storchova Z (2014). Ploidy changes and genome stability in yeast. Yeast.

[35] Chin CS, Peluso P, Sedlazeck FJ, Nattestad M, Concepcion GT, Clum A (2016). Phased diploid genome assembly with single-molecule real-time sequencing. Nat Methods.

[36] Schwessinger B, Sperschneider J, Cuddy WS, Garnica DP, Miller ME, Taylor JM (2018). A near-complete haplotype-phased genome of the dikaryotic wheat stripe rust fungus *Puccinia striiformis* f. sp. *tritici* reveals high interhaplotype diversity. MBio..

[37] Mondo SJ, Dannebaum RO, Kuo RC, Louie KB, Bewick AJ, LaButti K (2017). Widespread adenine N6-methylation of active genes in fungi. Nat Genet.

[38] Marçais G, Delcher AL, Phillippy AM, Coston R, Salzberg SL, Zimin A (2018). MUMmer4: a fast and versatile genome alignment system. PLoS Comput Biol.

[39] Shen XX, Opulente DA, Kominek J, Zhou X, Steenwyk JL, Buh KV (2018). Tempo and mode of genome evolution in the budding yeast subphylum. Cell.

[40] Enright AJ, Van Dongen S, Ouzounis CA (2002). An efficient algorithm for large-scale detection of protein families. Nucleic Acids Res.

[41] Kelley LA, Mezulis S, Yates CM, Wass MN, Sternberg MJ (2015). The Phyre2 web portal for protein modeling, prediction and analysis. Nat Protoc.

[42] Reynolds CR, Islam SA, Sternberg MJE (2018). EzMol: a web server wizard for the rapid visualization and image production of protein and nucleic acid structures. J Mol Biol.

[43] Damm U, Fourie PH, Crous PW (2010). *Coniochaeta* (*Lecythophora*), *Collophora* gen. nov. and *Phaeomoniella* species associated with wood necroses of Prunus trees. Persoonia..

[44] Xie J, Strobel GA, Feng T, Ren H, Mends MT, Zhou Z (2015). An endophytic *Coniochaeta velutina* producing broad spectrum antimycotics. J Microbiol..

[45] Khan Z, Gené J, Ahmad S, Cano J, Al-Sweih N, Joseph L (2013). *Coniochaeta polymorpha*, a new species from endotracheal aspirate of a preterm neonate, and transfer of *Lecythophora* species to *Coniochaeta*. Antonie Van Leeuwenhoek.

[46] de Lima Brossi MJ, Jiménez DJ, Cortes-Tolalpa L, van Elsas JD (2016). Soil-derived microbial consortia enriched with different plant biomass reveal distinct players acting in lignocellulose degradation. Microb Ecol.

[47] Cortes-Tolalpa L, Jiménez DJ, de Lima Brossi MJ, Salles JF, van Elsas JD (2016). Different inocula produce distinctive microbial consortia with similar lignocellulose degradation capacity. Appl Microbiol Biotechnol.

[48] U’Ren JM, Lutzoni F, Miadlikowska J, Laetsch AD, Arnold AE (2012). Host and geographic structure of endophytic and endolichenic fungi at a continental scale. Am J Bot.

[49] Jiménez DJ, De Mares M, Salles JF (2018). Temporal expression dynamics of plant biomass-degrading enzymes by a synthetic bacterial consortium growing on sugarcane bagasse. Front Microbiol..

[50] Karnaouri A, Topakas E, Antonopoulou I, Christakopoulos P (2014). Genomic insights into the fungal lignocellulolytic system of *Myceliophthora thermophila*. Front Microbiol..

[51] Zhang J, Siika-Aho M, Tenkanen M, Viikari L (2011). The role of acetyl xylan esterase in the solubilization of xylan and enzymatic hydrolysis of wheat straw and giant reed. Biotechnol Biofuels.

[52] Fujita K, Takashi Y, Obuchi E, Kitahara K, Suganuma T (2014). Characterization of a novel β-l-arabinofuranosidase in *Bifidobacterium longum*: functional elucidation of a DUF1680 protein family member. J Biol Chem.

[53] Ito T, Saikawa K, Kim S, Fujita K, Ishiwata A, Kaeothip S (2014). Crystal structure of glycoside hydrolase family 127 β-l-arabinofuranosidase from *Bifidobacterium longum*. Biochem Biophys Res Commun.

[54] Beever RE, Parkes SL (2003). Use of nitrate non-utilising (Nit) mutants to determine vegetative compatibility in *Botryotinia fuckeliana* (*Botrytis cinerea*). Eur J Plant Pathol.

[55] Brooker NL, Leslie JF, Dickman MB (1991). Nitrate non-utilizing mutants of *Colletotrichum* and their use in studies of vegetative compatibility and genetic relatedness. Phytopathology..

[56] Dilokpimol A, Mäkelä MR, Aguilar-Pontes MV, Benoit-Gelber I, Hildén KS, de Vries RP (2016). Diversity of fungal feruloyl esterases: updated phylogenetic classification, properties, and industrial applications. Biotechnol Biofuels.

[57] Müller G, Várnai A, Johansen KS, Eijsink VG, Horn SJ (2015). Harnessing the potential of LPMO-containing cellulase cocktails poses new demands on processing conditions. Biotechnol Biofuels.

[58] Busk PK, Lange L (2015). Classification of fungal and bacterial lytic polysaccharide monooxygenases. BMC Genomics..

[59] O’Connell RJ, Thon MR, Hacquard S, Amyotte SG, Kleemann J, Torres MF (2012). Lifestyle transitions in plant pathogenic *Colletotrichum* fungi deciphered by genome and transcriptome analyses. Nat Genet.

[60] Johansen KS (2016). Lytic polysaccharide monooxygenases: the microbial power tool for lignocellulose degradation. Trends Plant Sci.

[61] Frandsen KE, Lo Leggio L (2016). Lytic polysaccharide monooxygenases: a crystallographer’s view on a new class of biomass-degrading enzymes. IUCrJ..

[62] Span EA, Suess DLM, Deller MC, Britt RD, Marletta MA (2017). The role of the secondary coordination sphere in a fungal polysaccharide monooxygenase. ACS Chem Biol.

[63] Nichols NN, Hector RE, Frazer SE (2019). Genetic transformation of *Coniochaeta* sp. 2T2.1, key fungal member of a lignocellulose-degrading microbial consortium. Biol Methods Protocols..

[64] Grabherr MG, Haas BJ, Yassour M, Levin JZ, Thompson DA, Amit I (2011). Full-length transcriptome assembly from RNA-Seq data without a reference genome. Nat Biotechnol.

[65] Grigoriev IV, Nikitin R, Haridas S, Kuo A, Ohm R, Otillar R (2014). MycoCosm portal: gearing up for 1000 fungal genomes. Nucleic Acids Res.

[66] Frazer KA, Pachter L, Poliakov A, Rubin EM, Dubchak I (2004). VISTA: computational tools for comparative genomics. Nucleic Acids Res.

[67] Kohler A, Kuo A, Nagy LG, Morin E, Barry KW, Buscot F (2015). Convergent losses of decay mechanisms and rapid turnover of symbiosis genes in mycorrhizal mutualists. Nat Genet.

[68] Haitjema CH, Gilmore SP, Henske JK, Solomon KV, de Groot R, Kuo A (2017). A parts list for fungal cellulosomes revealed by comparative genomics. Nat Microbiol..

[69] Nazareno ES, Li F, Smith M, Park RF, Kianian SF, Figueroa M (2018). *Puccinia coronata* f. sp. *avenae*: a threat to global oat production. Mol Plant Pathol..

[70] Murat C, Kuo A, Barry KW, Clum A, Dockter RB, Fauchery L (2018). Draft genome sequence of *Tuber borchii* Vittad, a Whitish Edible Truffle. Genome Announc..

[71] Pomraning KR, Bredeweg EL, Kerkhoven EJ, Barry K, Haridas S, Hundley H (2018). Regulation of yeast-to-hyphae transition in *Yarrowia lipolytica*. mSphere..

[72] Vesth TC, Nybo JL, Theobald S, Frisvad JC, Larsen TO, Nielsen KF (2018). Investigation of inter- and intraspecies variation through genome sequencing of *Aspergillus* section Nigri. Nat Genet.

[73] Urquhart AS, Mondo SJ, Mäkelä MR, Hane JK, Wiebenga A, He G (2018). Genomic and genetic insights into a cosmopolitan fungus, *Paecilomyces variotii* (Eurotiales). Front Microbiol..

[74] Mondo SJ, Salvioli A, Bonfante P, Morton JB, Pawlowska TE (2016). Nondegenerative evolution in ancient heritable bacterial endosymbionts of fungi. Mol Biol Evol.

[75] Edgar RC (2004). MUSCLE: multiple sequence alignment with high accuracy and high throughput. Nucleic Acids Res.

[76] Suyama M, Torrents D, Bork P (2006). PAL2NAL: robust conversion of protein sequence alignments into the corresponding codon alignments. Nucleic Acids Res.

[77] Yang ZH, Nielsen R (2000). Estimating synonymous and nonsynonymous substitution rates under realistic evolutionary models. Mol Biol Evol.

[78] Yang Z (2007). PAML 4: phylogenetic analysis by maximum likelihood. Mol Biol Evol.

[79] Wang Y, Coleman-Derr D, Chen G, Gu YQ (2015). OrthoVenn: a web server for genome wide comparison and annotation of orthologous clusters across multiple species. Nucleic Acids Res.

[80] Huang L, Zhang H, Wu P, Entwistle S, Li X, Yohe T (2018). dbCAN-seq: a database of carbohydrate-active enzyme (CAZyme) sequence and annotation. Nucleic Acids Res.

[81] Katoh K, Toh H (2008). Improved accuracy of multiple ncRNA alignment by incorporating structural information into a MAFFT-based framework. BMC Bioinform.

[82] Talavera G, Castresana J (2007). Improvement of phylogenies after removing divergent and ambiguously aligned blocks from protein sequence alignments. Syst Biol.

[83] Price MN, Dehal PS, Arkin AP (2010). FastTree 2–approximately maximum-likelihood trees for large alignments. PLoS ONE.

[84] Stamatakis A (2014). RAxML version 8: a tool for phylogenetic analysis and post-analysis of large phylogenies. Bioinformatics.

[85] Lombard V, Golaconda Ramulu H, Drula E, Coutinho PM, Henrissat B (2014). The carbohydrate-active enzymes database (CAZy) in 2013. Nucleic Acids Res.

[86] Nielsen H (2017). Predicting secretory proteins with signalP. Methods Mol Biol.

[87] Kim D, Langmead B, Salzberg SL (2015). HISAT: a fast spliced aligner with low memory requirements. Nat Methods.

[88] Liao Y, Smyth GK, Shi W (2014). featureCounts: an efficient general purpose program for assigning sequence reads to genomic features. Bioinformatics.

[89] Love MI, Huber W, Anders S (2014). Moderated estimation of fold change and dispersion for RNA-seq data with DESeq2. Genome Biol.

