# Supplementary material for: Genome expansion by allopolyploidization in the fungal strain Coniochaeta 2T2.1 and its exceptional lignocellulolytic machinery
Source: Biotechnol Biofuels. 2019 Sep 23;12:229. doi: 10.1186/s13068-019-1569-6 (PMC6757388; doi:10.1186/s13068-019-1569-6)
Supplement: Supplementary file 3 — Additional file 3. Additional figures (S1 to S3). [file 13068_2019_1569_MOESM3_ESM.pptx]

## Slide 1
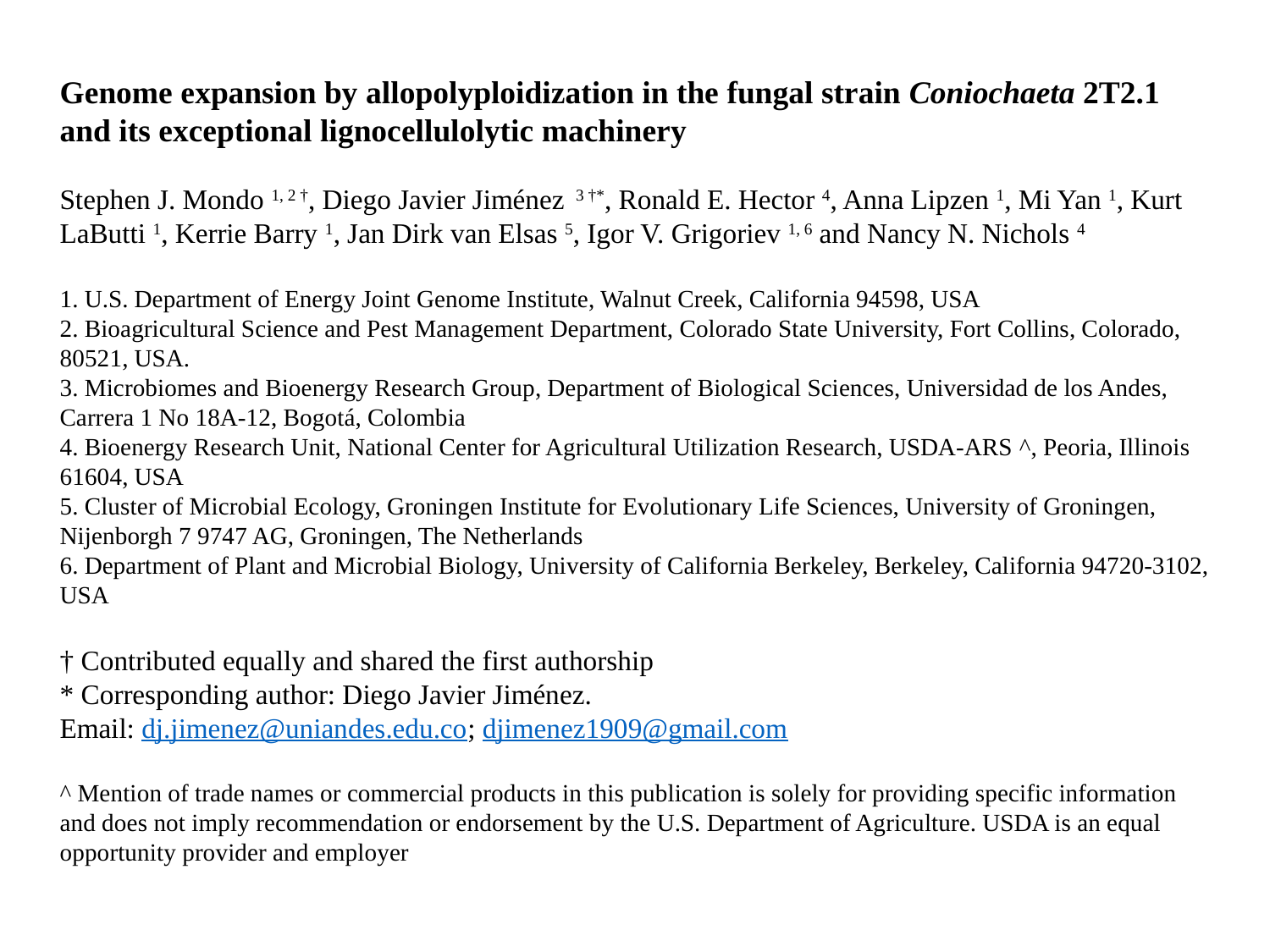

Genome expansion by allopolyploidization in the fungal strain Coniochaeta 2T2.1 and its exceptional lignocellulolytic machinery
Stephen J. Mondo 1, 2 †, Diego Javier Jiménez 3 †*, Ronald E. Hector 4, Anna Lipzen 1, Mi Yan 1, Kurt LaButti 1, Kerrie Barry 1, Jan Dirk van Elsas 5, Igor V. Grigoriev 1, 6 and Nancy N. Nichols 4
1. U.S. Department of Energy Joint Genome Institute, Walnut Creek, California 94598, USA
2. Bioagricultural Science and Pest Management Department, Colorado State University, Fort Collins, Colorado, 80521, USA.
3. Microbiomes and Bioenergy Research Group, Department of Biological Sciences, Universidad de los Andes, Carrera 1 No 18A-12, Bogotá, Colombia
4. Bioenergy Research Unit, National Center for Agricultural Utilization Research, USDA-ARS ^, Peoria, Illinois 61604, USA
5. Cluster of Microbial Ecology, Groningen Institute for Evolutionary Life Sciences, University of Groningen, Nijenborgh 7 9747 AG, Groningen, The Netherlands
6. Department of Plant and Microbial Biology, University of California Berkeley, Berkeley, California 94720-3102, USA
† Contributed equally and shared the first authorship
* Corresponding author: Diego Javier Jiménez.
Email: dj.jimenez@uniandes.edu.co; djimenez1909@gmail.com
^ Mention of trade names or commercial products in this publication is solely for providing specific information and does not imply recommendation or endorsement by the U.S. Department of Agriculture. USDA is an equal opportunity provider and employer

## Slide 2
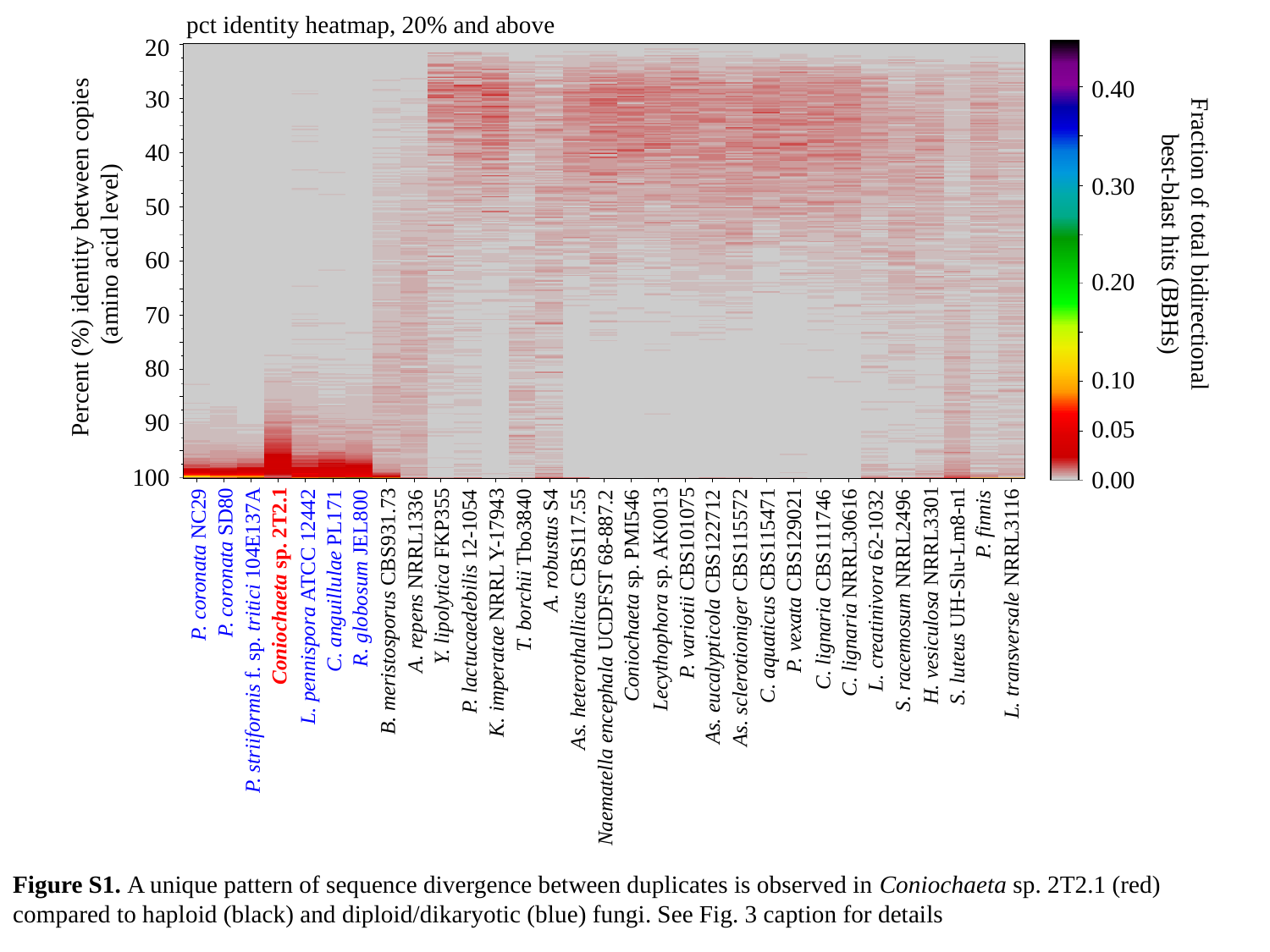

pct identity heatmap, 20% and above
20
0.40
30
40
0.30
50
Fraction of total bidirectional best-blast hits (BBHs)
Percent (%) identity between copies
(amino acid level)
60
0.20
70
80
0.10
90
0.05
100
0.00
P. finnis
A. robustus S4
P. coronata SD80
P. coronata NC29
T. borchii Tbo3840
Y. lipolytica FKP355
P. vexata CBS129021
A. repens NRRL1336
R. globosum JEL800
C. anguillulae PL171
P. variotii CBS101075
C. lignaria CBS111746
Coniochaeta sp. 2T2.1
C. lignaria NRRL30616
L. creatinivora 62-1032
Coniochaeta sp. PMI546
C. aquaticus CBS115471
S. luteus UH-Slu-Lm8-n1
H. vesiculosa NRRL3301
S. racemosum NRRL2496
Lecythophora sp. AK0013
L. transversale NRRL3116
P. lactucaedebilis 12-1054
L. pennispora ATCC 12442
B. meristosporus CBS931.73
K. imperatae NRRL Y-17943
As. eucalypticola CBS122712
As. sclerotioniger CBS115572
As. heterothallicus CBS117.55
P. striiformis f. sp. tritici 104E137A
Naematella encephala UCDFST 68-887.2
Figure S1. A unique pattern of sequence divergence between duplicates is observed in Coniochaeta sp. 2T2.1 (red) compared to haploid (black) and diploid/dikaryotic (blue) fungi. See Fig. 3 caption for details

## Slide 3
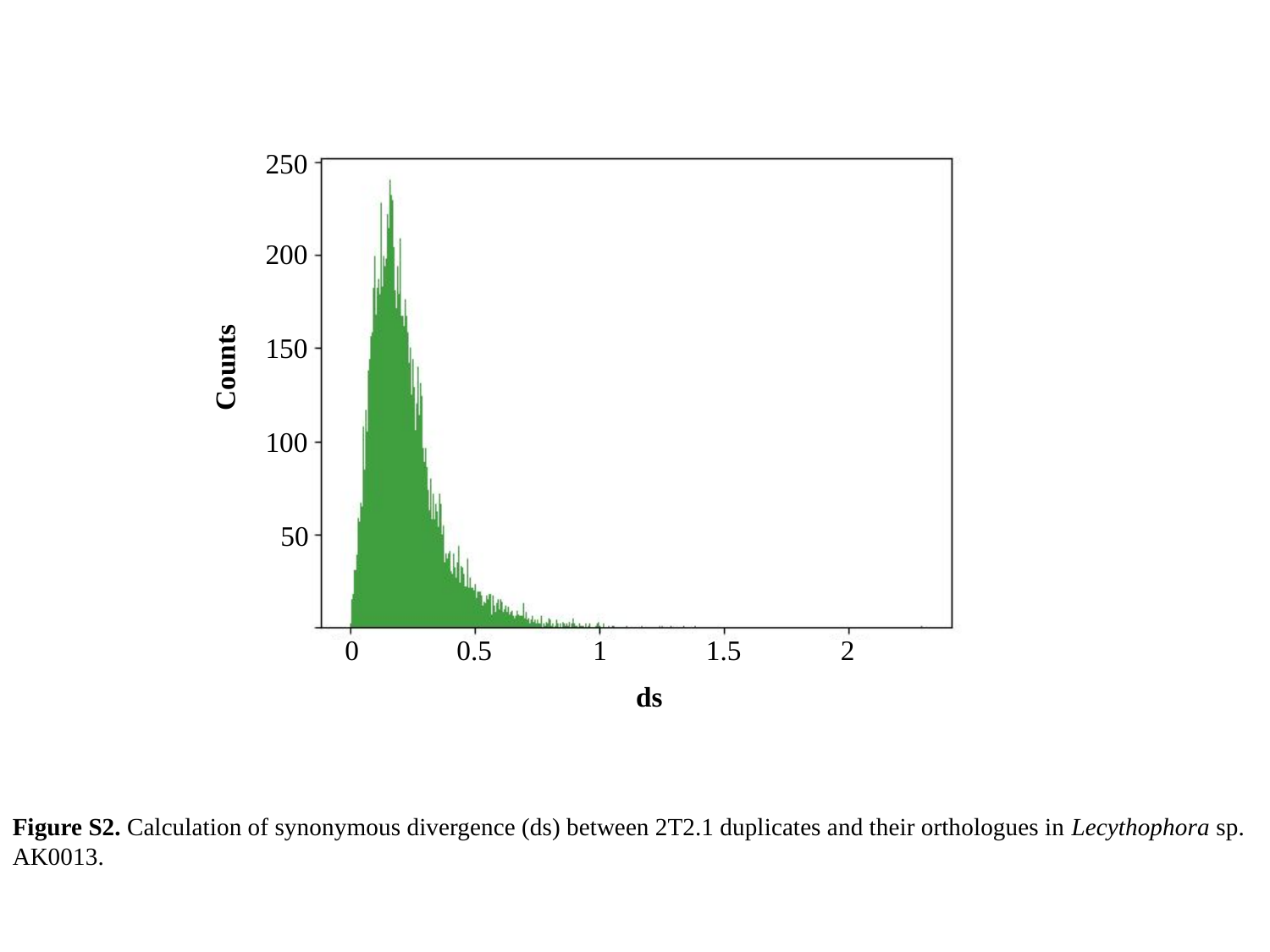

250
200
150
Counts
100
50
0.5
1
1.5
2
0
ds
Figure S2. Calculation of synonymous divergence (ds) between 2T2.1 duplicates and their orthologues in Lecythophora sp. AK0013.

## Slide 4
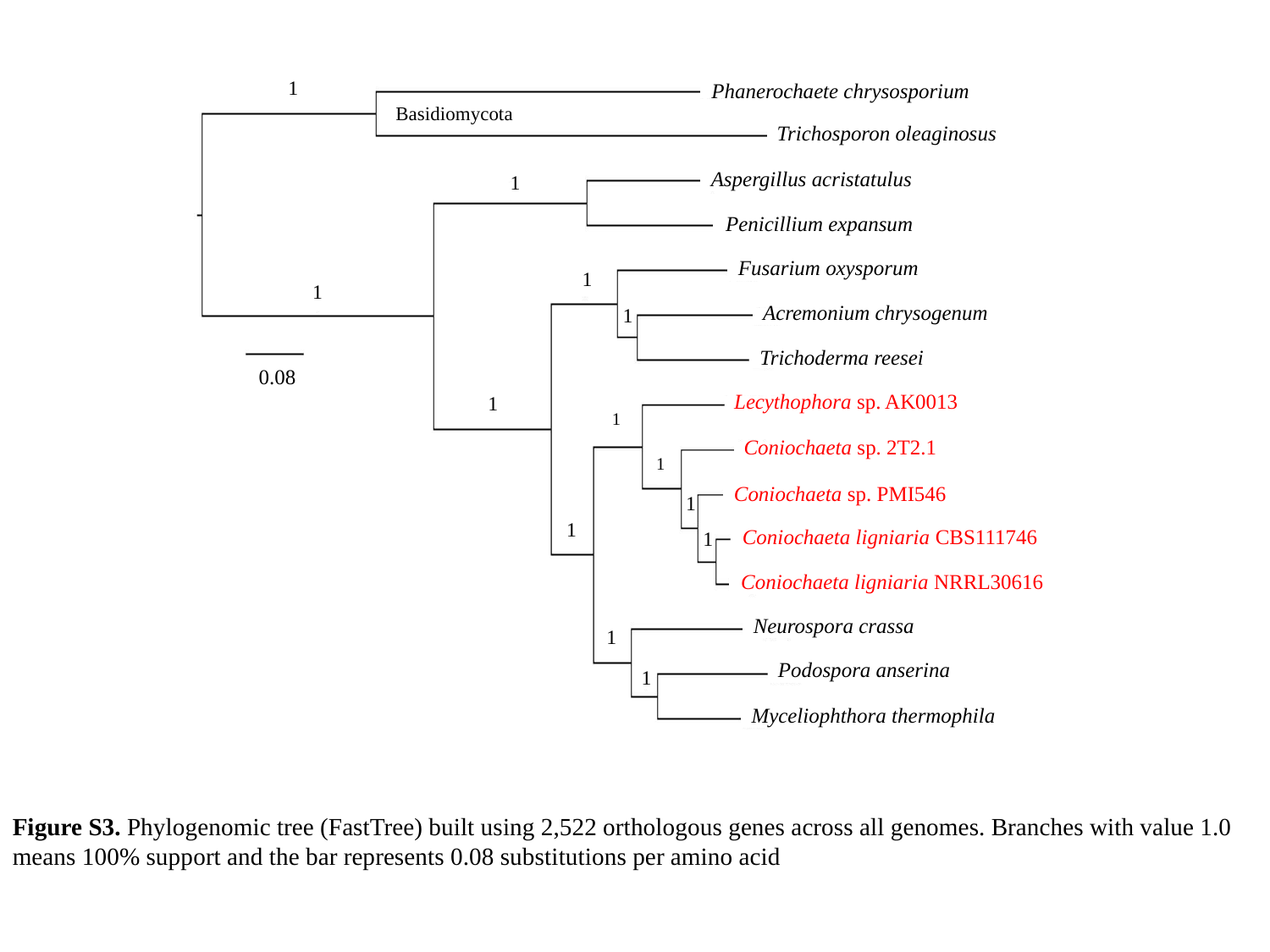

1
Phanerochaete chrysosporium
Basidiomycota
Trichosporon oleaginosus
Aspergillus acristatulus
1
Penicillium expansum
Fusarium oxysporum
1
1
Acremonium chrysogenum
Trichoderma reesei
0.08
Lecythophora sp. AK0013
1
1
Coniochaeta sp. 2T2.1
1
Coniochaeta sp. PMI546
1
Coniochaeta ligniaria CBS111746
Coniochaeta ligniaria NRRL30616
Neurospora crassa
1
Podospora anserina
Myceliophthora thermophila
1
1
1
1
Figure S3. Phylogenomic tree (FastTree) built using 2,522 orthologous genes across all genomes. Branches with value 1.0 means 100% support and the bar represents 0.08 substitutions per amino acid

## Slide 5
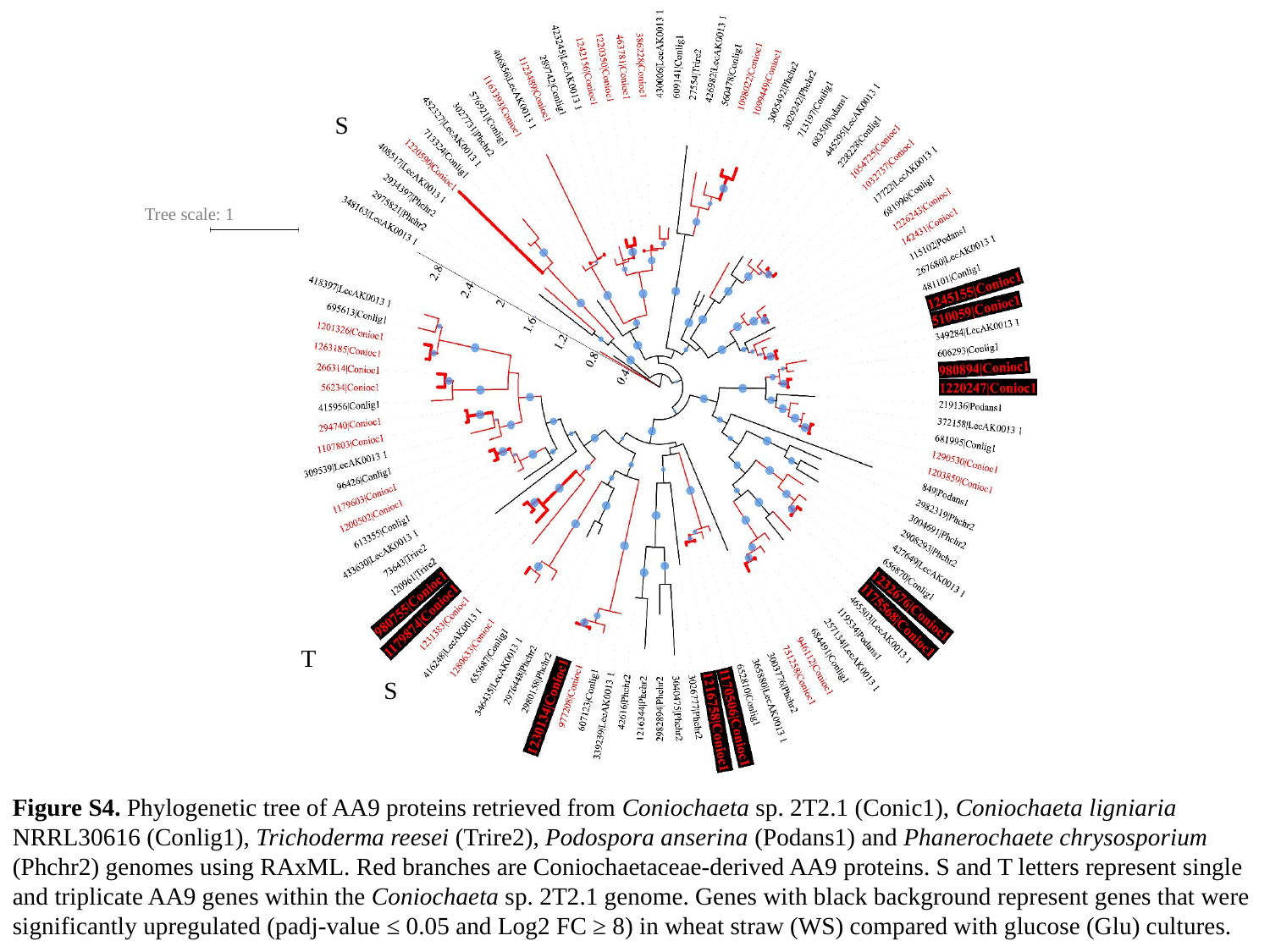

S
Tree scale: 1
T
S
Figure S4. Phylogenetic tree of AA9 proteins retrieved from Coniochaeta sp. 2T2.1 (Conic1), Coniochaeta ligniaria NRRL30616 (Conlig1), Trichoderma reesei (Trire2), Podospora anserina (Podans1) and Phanerochaete chrysosporium (Phchr2) genomes using RAxML. Red branches are Coniochaetaceae-derived AA9 proteins. S and T letters represent single and triplicate AA9 genes within the Coniochaeta sp. 2T2.1 genome. Genes with black background represent genes that were significantly upregulated (padj-value ≤ 0.05 and Log2 FC ≥ 8) in wheat straw (WS) compared with glucose (Glu) cultures.
