# Supplementary material for: Genome expansion by allopolyploidization in the fungal strain Coniochaeta 2T2.1 and its exceptional lignocellulolytic machinery
Source: Biotechnol Biofuels. 2019 Sep 23;12:229. doi: 10.1186/s13068-019-1569-6 (PMC6757388; doi:10.1186/s13068-019-1569-6)
Supplement: Supplementary file 7 — Additional file 7: Table S6. 3D modeling features of five selected AA9 proteins that were significantly and highly upregulated (padj-value ≤ 0.05 and Log2 FC ≥ 8) on wheat straw (WS) compared with glucose (Glu) cultures. [file 13068_2019_1569_MOESM7_ESM.docx]

**Additional file 7. Table S5.** 3D modeling features of five selected AA9 proteins that were significantly and highly upregulated (padj-value ≤ 0.05 and Log2 FC ≥ 8) on wheat straw (WS) compared with glucose (Glu) cultures.

| **JGI-IDs (aa)** | **kDA/IP** | **Model dimensions (Å)** | **Confidence and coverage *** | **Best protein for modeling (PDB-ID) {% identity} * [Reference]** |
| --- | --- | --- | --- | --- |
| 1170506 (240) | 23.76 / 7.51 | X:38.000 Y:49.272 Z:49.612 | 92% of residues modelled at >90% confidence | Structural basis for substrate targeting and catalysis by fungal polysaccharide monooxygenases PMO-3 (4EIS) {74%}  [1] |
| 980755 (241) | 23.96 / 4.56 | X:41.278 Y:58.648 Z:47.700 | 93% of residues modelled at >90% confidence | Extended catalytic domain of *Hypocrea jecorina* LPMO9A a.k.a EG4 (5O2X) {49} [2] |
| 1220247 (309) | 29.81 / 6.99 | X:56.620 Y:56.590 Z:46.809 | 79% of residues modelled at >90% confidence | Crystal structure of the catalytic domain of NcLPMO9A (5FOH) {41} [unpublished] |
| 1175568 (227) | 22.17 / 7.13 | X:35.554 Y:38.838 Z:54.548 | 91% of residues modelled at >90% confidence | Magnesium-bound glycoside hydrolase 61 isoform E from *Thielavia terrestris* (3EJA) {63} [3] |
| 1230134 (255) | 25.05 / 6.96 | X:38.653 Y:45.812 Z:55.149 | 90% of residues modelled at >90% confidence | Crystal Structure of a Cellulose-active Polysaccharide Monooxygenase from *Myceliophthora thermophila* MtPMO3 (5UFV) {80} [4] |

aa: Number of amino acids; kDa: KiloDaltons; IP: Isoelectric point

* Data obtained by using the Phyre2 portal (intensive modelling) [5]

**References**

1. Li X, Beeson WT, Phillips CM, Marletta MA, Cate JH. Structural basis for substrate targeting and catalysis by fungal polysaccharide monooxygenases. Structure. 2012;20:1051-1061.

2. Hansson H, Karkehabadi S, Mikkelsen N, Douglas NR, Kim S, Lam A, et al. High-resolution structure of a lytic polysaccharide monooxygenase from *Hypocrea jecorina* reveals a predicted linker as an integral part of the catalytic domain. J Biol Chem. 2017;292:19099-19109.

3. Harris PV, Welner D, McFarland KC, Re E, Navarro Poulsen JC, Brown K, et al. Stimulation of lignocellulosic biomass hydrolysis by proteins of glycoside hydrolase family 61: structure and function of a large, enigmatic family. Biochemistry. 2010;49:3305-3316.

4. Span EA, Suess DLM, Deller MC, Britt RD, Marletta MA. The Role of the Secondary Coordination Sphere in a Fungal Polysaccharide Monooxygenase. ACS Chem Biol. 2017;12:1095-1103.

5. Kelley LA, Mezulis S, Yates CM, Wass MN, Sternberg MJ. The Phyre2 web portal for protein modeling, prediction and analysis. Nat Protoc. 2015;10:845-858.
